# The “multiple exposure effect” (MEE): How multiple exposures to similarly biased online content can cause increasingly larger shifts in opinions and voting preferences

**DOI:** 10.1371/journal.pone.0322900

**Published:** 2025-05-12

**Authors:** Robert Epstein, Amanda Newland, Li Yu Tang

**Affiliations:** American Institute for Behavioral Research and Technology, Vista, California, United States of America; Roma Tre University: Universita degli Studi Roma Tre, ITALY

## Abstract

In three randomized, controlled experiments performed on simulations of three popular online platforms – Google search, X/Twitter, and Alexa – with a total of 1,488 undecided, eligible US voters, we asked whether multiple exposures to similarly biased content on those platforms could shift opinions and voting preferences more than a single exposure could. All participants were first shown brief biographies of two political candidates, then asked about their voting preferences, then exposed to biased content on one of our three simulated platforms, and then asked again about their voting preferences. In all experiments, participants in different groups saw biased content favoring one candidate, his or her opponent, or neither. In all the experiments, our primary dependent variable was Vote Manipulation Power (VMP), the percentage increase in the number of participants inclined to vote for one candidate after having viewed content favoring that candidate. In Experiment 1 (on our Google simulator), the VMP increased with successive searches from 14.3% to 20.2% to 22.6%. In Experiment 2 (on our X/Twitter simulator), the VMP increased with successive exposures to biased tweets from 49.7% to 61.8% to 69.1%. In Experiment 3 (on our Alexa simulator), the VMP increased with successive exposures to biased replies from 72.1% to 91.2% to 98.6%. Corresponding shifts were also generally found for how much participants reported liking and trusting the candidates and for participants’ overall impression of the candidates. Because multiple exposures to similarly biased content might be common on the internet, we conclude that our previous reports about the possible impact of biased content – always based on single exposures – might have underestimated its possible impact. Findings in our new experiments exemplify what we call the “multiple exposure effect” (MEE).

## 1 Introduction

The internet has made possible a number of new forms of influence, some of which are largely invisible to users. Most rely on ephemeral content – content that is presented briefly, has an impact on the user, and then disappears – which allows these forms of influence to impact people without leaving a paper trail for authorities to trace [1–3]. Most of these new forms of influence are controlled exclusively by a handful of tech monopolies, which means they cannot be counteracted; when an algorithm controlled by a large tech company favors one brand of guitar or one political candidate, opponents have no way to counteract the impact of that algorithm. Content posted by opponents might even be suppressed by that algorithm. When online content is suppressed, injured parties generally have no recourse, even in the courts [4–7].

An ever-growing group of scholars and scientists has expressed concern over the power that online companies have to influence people’s thinking and behavior [8–10], and these concerns have recently been amplified by the online deployment of highly capable AI applications such as ChatGPT [11–14]. In 2023, two public petitions attracted signatures from tens of thousands of experts in computer science and related fields expressing concerns about AI – the first calling for governments worldwide to “step in and institute a moratorium” if AI labs refuse to pause research [13] and the second expressing explicit concern about the possibility that emerging AI technology could lead to the extinction of humankind and should be made a “global priority” alongside “pandemics and nuclear war” [14].

Since 2013, our research group has been identifying, studying, and quantifying new forms of influence that the internet has made possible [15–24]. The first of these was the Search Engine Manipulation Effect (SEME) [15], an effect that has been replicated fully or partially multiple times [16,25–36] and that was anticipated by an earlier study in which bias in search results was shown to impact people’s views about the value of vaccinations [37]. SEME studies show that when people who are undecided on some issue are exposed to biased search results – that is to say, a list of search results in which higher-ranked results link to web pages that support one point of view – the opinions of those people about that issue can shift substantially after just one search. The increase in the proportion of people favoring one point of view has been shown to increase by as much as 80% in some demographic groups [15].

SEME appears to be one of the largest effects ever discovered in the behavioral sciences, perhaps because, unlike other list effects, it is supported by a daily regimen of operant conditioning [27]. When people hear someone recite a list of words, they are likely to remember the first and last words on the list best – a phenomenon called the “serial position effect.” The only thing that differentiates those words, however, is their position on the list. A list of search results is different, because users are taught, day after day, to value higher-ranking search results over lower-ranking ones. This is because about 83% [38] of the searches people conduct are for the answers to simple factual questions (“What is the capitol of Idaho?”), and the answers to those questions invariably turn up in the highest search result. People thus learn, over and over again, that higher-ranked results are better than lower ones. Because this bias is so strong, when users submit open-ended queries (“What is the best restaurant in Chicago?”), they trust webpages linked to high-ranking search results. That repeatedly conditioned trust might explain why SEME is such a large effect.

In articles that have not been peer reviewed, one author opined that VMP is not a good measure of the impact of biased search results [39], and others have claimed that bias in search results does not exist [40–42]. Still others have noted that bias in search results as a source of influence might easily be overwhelmed by the many other sources of influence to which consumers and voters are exposed [43]. The question about whether political bias exists in search results is beyond the scope of this paper (although see [44–61]). We submit, however, that the size of SEME we measured in controlled experiments might in fact have *underestimated* the possible size of the effect in the real world.

We believe this may be true because in previous SEME experiments, people were exposed to biased search results just once, typically for a maximum of 15 min. In the real world, a search result platform might, over time, present users with similarly biased search results dozens or even hundreds of times. The same can be said of other new forms of influence we have studied, such as the “answer bot effect” (ABE) [17], the “targeted messaging effect” (TME) [18], the “search suggestion effect” (SSE) [19], and the “video manipulation effect” (VME) [22]. We establish such effects with just a single exposure to biased content on one platform. In the real world, however, people might be exposed to similarly biased content on the same online platform many times. We call the cumulative impact of multiple exposures to similarly biased online content the “multiple exposure effect” (MEE). We speculate that the cumulative impact is additive, and we investigate that possibility in the present study. We distinguish MEE from other repeated exposure effects in only one major respect, namely that it applies to similarly repeated exposure to similarly biased content on the internet. Most research on repeated exposure has looked at content researchers studied long before the internet began to dominate people’s lives.

### 1.1 The impact of repeated exposures

The impact of repeated exposures to a stimulus or setting has been studied for more than a century, beginning, perhaps, with Ebbinghaus’ experiments on his own memory [62], Thorndike’s puzzle box experiments [63], and Pavlov’s early experiments on classical conditioning [64]. Generally speaking, repeated exposure to an association that can be learned – to a pairing of the sound of a bell, for example, with the delivery of food – improves learning [64]. Repeated exposure to an innocuous stimulus such as the sound of traffic typically leads to adaptation – to a decrease in awareness, sometimes to a point at which awareness disappears [65]. And repeated exposure to a noxious stimulus such as an electric shock can, depending on the type and magnitude of that stimulus, lead either to adaptation or to sensitization – a sometimes dramatic increase in the magnitude of the reaction to the stimulus [66].

In recent decades, behavioral scientists who have studied repeated exposure have shown, among other things, that the repeated presentation of photographs of faces generally increases how attractive people find those faces [67]; that repeated exposure to the taste of fruits and vegetables generally increases preferences for those foods by young children [68]; that repeated exposure to neutral or mildly pleasant odors generally increases appreciation for those odors [69]; and that repeated exposure to unfamiliar tonal and nontonal melodies increases liking ratings for those melodies [70,71]. In general, stimuli that are presented repeatedly are rated more positively than novel stimuli; this effect – often called the “mere exposure effect” [72] – is especially important in fields such as advertising [73–76]. The mere exposure effect has been demonstrated “even when exposures are not accessible to awareness” [77].

Persuasive messages have also been shown to be more effective with repetition [78,79]; in general, people believe information that is repeated more than they believe novel information – a finding called “the truth effect” [80–84]. It is especially relevant to the present investigation that the truth effect has been demonstrated even when the repeated information is false [81,84].

The internet has dramatically increased the frequency with which the same information – both true and false – can be presented to billions of people over short periods of time [85,86]. Unfortunately, misinformation about important issues, such as risk factors associated with the spread of COVID-19, can rapidly spread faulty beliefs about such matters, along with anxieties associated with such beliefs [85]. Repetition of “fake news” stories on the internet has been shown to strengthen people’s beliefs even in relatively absurd stories as long as they have even “a small degree of potential plausibility” [86; cf. 87,88]. One recent study of “plausibility boundaries” concludes, sadly, that “[w]hen the truth is hard to come by, fluency is an attractive stand-in” [86].

Online repetition of political content – even what one recent study calls “brute force” activity levels of political content on platforms such as Facebook and Twitter [89] – can have a substantial impact on voters. “An increase in one standard deviation of [Facebook] resonance…” – defined by the authors as “other users liking, commenting on and sharing posts by candidates” – “translates into an average of some additional 1213 votes” [89; cf. 90–93].

Repetition of subtle online manipulations has been shown to increase the impact of those manipulations. In one of our recent experiments on the “answer bot effect” (ABE), for example, a single question-and-answer interaction on an Alexa simulator was shown to produce a shift of voting preferences among undecided voters of 43.8%; when undecided voters were exposed to six similarly biased answers on our simulator, the shift increased to 65.8% [17]. Although the repetition increased the shift in voting preferences, it also increased awareness of possible bias in the answers; with one interaction, 4.9% of participants speculated that the answer given might be biased; that percentage increased to 40.7% for participants exposed to six similarly biased answers [17].

In a recent study on the “targeted messaging effect” (TME), in which a political candidate was disparaged in a single negative tweet embedded among 30 neutral tweets sent to study participants (all undecided voters), voting preferences shifted toward the opposing candidate by 32.4%. For participants (again, all undecided voters) who were exposed to five negative tweets embedded among 30 neutral tweets, voting preferences shifted toward the opposing candidate by 87.0% [18]. Once again, when the biased content was repeated, awareness of possible bias was higher (2.1% of participants) than when such content was presented only once (0.8% of participants) [18].

In the present investigation, we expand the existing and growing literature on online repetition by measuring the effect of repeatedly presenting people with similarly biased content on simulations of two popular online platforms – Google search and X (f.k.a. Twitter) – as well as on our simulation of Alexa, a popular intelligent personal assistant (IPA).

## 2 Experiment 1. Multiple exposures to similarly biased content on a search engine simulator

### 2.1 Methods

#### 2.1.1 Ethics statement.

The federally registered Institutional Review Board (IRB) of the sponsoring institution (American Institute for Behavioral Research and Technology) approved this study with exempt status under HHS rules because (a) the anonymity of participants was preserved and (b) the risk to participants was minimal. The IRB is registered with OHRP under number IRB00009303, and the Federalwide Assurance number for the IRB is FWA00021545. Informed written consent was obtained for all experiments as specified in the Procedure section below. We also confirm that all experiments were performed in accordance with relevant guidelines and regulations. Our methods were not preregistered.

#### 2.1.2 Participants.

Participants were recruited online from the Amazon Mechanical Turk (MTurk) subject pool between May 9 and 10, 2016 [94–96]. This was well before concerns began to be raised about the growing number of bots in that subject pool [97]. Participants had to be residents of the US and at least 18 years old. They were not allowed to proceed if they answered Yes to the following pre-screening question: “Have you already decided who you will vote for in the upcoming US presidential election?” This screening assured, or at least increased the likelihood, that our participants were vulnerable to being impacted by our experimental manipulation.

Before cleaning we had data from 857 participants. We removed participants who reported an age lower than 18, reported residing outside of the US, and duplicate cases. We also asked participants to report their English fluency on a scale from 1 to 10, where 1 was labeled “Not fluent” and 10 was labeled “Highly fluent.” We removed participants who reported a fluency level below 6. Finally, we removed participants who exited from the experiment without clicking on any webpages. After cleaning we had data from 801 participants. To equalize the size of the six groups we analyzed, we used SPSS to draw at random (and without replacement) the largest possible sample we could obtain from each of the six groups, giving us 88 people in each. In total, therefore, we analyzed data from 528 people in this experiment.

The participants ranged in age from 18 to 70 (*M* = 32.8, median = 30, *SD* = 10.4). 46.4% (*n =* 245) of the participants identified themselves as male and 53.6% (*n =* 283) as female. 76.1% (*n =* 402) of the participants identified themselves as White, 6.6% (*n =* 35) as Hispanic, 6.6% (*n =* 35) as Asian, 6.0% (*n =* 31) as Black, 3.4% (*n =* 18) as mixed, and 1.3% (*n =* 7) as other. Regarding the level of education people reported having completed, 9.8% (*n =* 52) said high school, 41.0% (*n =* 216) said “some college,” 34.8% (*n =* 184) said bachelors degree, 10.6% (*n =* 56) said masters degree, and 3.8% (*n =* 20) said doctorate. 38.3% (*n =* 202) of the participants identified themselves as liberal, 37.8% (*n =* 200) as moderate, 14.8% (*n =* 78) as conservative, 6.8% (*n =* 36) as none, and 2.3% (*n =* 12) as other.

We also asked participants to rank how familiar they were with each candidate on a scale from 1 to 10 (where 1 was labeled “Not at all” and 10 was labeled “Quite familiar”). The mean familiarity score for Donald Trump was 8.06 (*SD* = 1.9), and the mean familiarity score for Hillary Clinton was 8.02 (*SD* = 2.0).

#### 2.1.3 Procedure.

Participants were given brief instructions and then asked for their informed consent to continue (S1 Text), after which they were asked basic demographic questions. As required by the sponsoring institution’s IRB, participants were not asked for identifying information such as name, email address, or telephone number. They were then shown brief biographies of the candidates that were identical in format; each was about 140 words in length (S2 Text).

The experiment used a pre/post design in which all participants were first asked a series of questions (pre-manipulation), then subjected to a manipulation (a search engine search), and then asked those same questions again (post-manipulation). After viewing the brief biographies, participants were asked eight questions about the candidates (the pre-manipulation questions). For each candidate they were first asked (on scales from 1 to 10, where 10 was highest) how much they liked and trusted that candidate, and how favorable their overall impression was of that candidate (S1 Fig). Then, on an 11-point scale, where values ranged from 5 to 0–5, they were asked to indicate which candidate they would vote for if they had to “vote today” (S2 Fig). The names of the candidates at either end of the scale were counterbalanced. Finally, they were asked which candidate they would vote for if they had to vote “right now” (forced choice) (S2 Fig).

**Fig 1:**
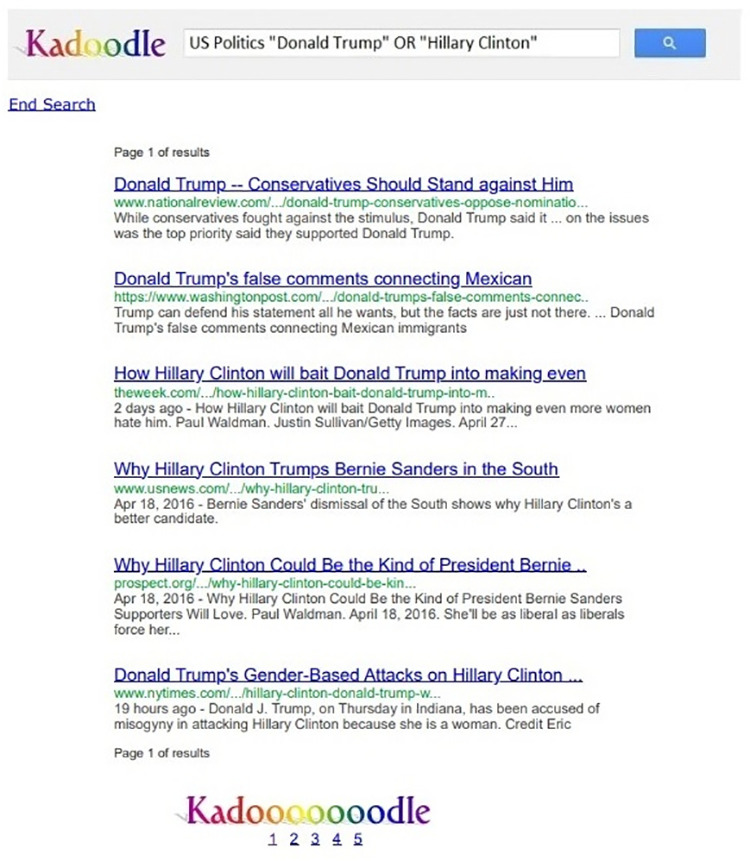
Experiment 1: Example of Kadoodle search results page. The search phrase in the search bar was pre-filled. Each of the five pages of search results included a list of six search results. The order of the results was different for each of the three groups. The order could favor Hillary Clinton (as shown above), Donald Trump, or neither Presidential candidate. See text for details. Reprinted from [26] under a CC BY license, with permission from the American Institute from Behavioral Research and Technology, original copyright 2024. This figure is similar but not identical to the original image and is therefore for illustrative purposes only.

**Fig 2:**
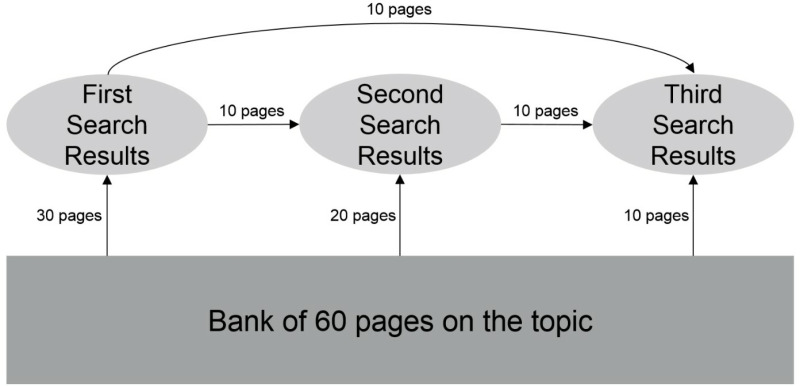
Experiment 1: Selection and grouping of search results for each of the three exposures. In the first exposure in multiple exposure conditions, 30 search results and corresponding web pages were selected from the total bank of 60. In the second exposure, 10 search results were taken from the first batch and combined with 20 new search results from the bank. The third exposure used 10 search results from the first batch (that had not been used in the second batch), 10 results from the second batch (that had not been used in the first batch), and the remaining 10 results from the bank (that had not previously been used in either of the first two batches).

They were then given up to 15 minutes to use Kadoodle, our Google simulator [15] to search for information about each candidate. Kadoodle looked and functioned almost exactly like Google. Unlike Google, it showed only five pages of search results with six results per page (Fig 1), but participants could click on any search result to view the corresponding web page, and they could also switch to different pages of search results by clicking on numbers at the bottom of each page of search results. Note that in all exposure conditions, participants had access to a total of 30 search results and 30 corresponding web pages; those 30 search results were drawn from a total pool of 60 search results and corresponding web pages (Fig 2).

All 60 web pages used in the study (in both Experiments 1 and 2) had previously been rated for bias by five independent people who rated each web page on a scale from 5 to 0–5, where “pro-Trump” appeared at one end of the scale and “pro-Clinton” appeared at the other end, with the order counterbalanced. Based on the mean bias ratings, the 60 web pages were ranked from the most pro-Trump to the most pro-Clinton, with relatively neutral (near 0 in mean bias) web pages in the middle.

Both the search results and web pages were real web content that had been sourced from the Google search engine and the internet. The web pages were image files we had created from the original HTML pages so that they contained no active links. The text in the search bar was pre-filled with the text, “US Politics ‘Donald Trump’ OR ‘Hillary Clinton’” (Fig 1).

On pages of search results, a button appeared in the upper-left corner of the screen reading “End Search” (Fig 1). When participants clicked that button, or when the 15-min search period ended (whichever came first), the search session ended, and the participants were again presented with the six opinion questions and two voting questions to which they had responded before the search (the post-manipulation questions). After they responded to the second set of questions, they were asked to indicate whether any of the content they had seen in the search had “bothered” them. If they clicked “yes,” they could then explain their answer at length in a text box. We asked whether content bothered them as a way of determining whether they had noticed any bias in the ordering of the search results they had seen (see below for details about that bias). We could not ask them directly about “bias,” because leading questions of that sort have long been known to inflate and distort answers [98].

**Fig 3:**
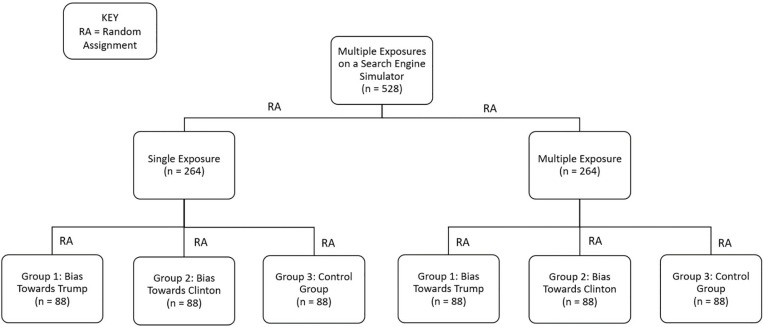
Experiment 1: Single and multiple exposure bias groups using a search engine. Participants were first split into either the single exposure or multiple exposure conditions. In the single exposure condition, they were then randomly assigned to either a pro-Trump, pro-Clinton, or control group (see text and Fig 4 for details). The same occurred in the multiple exposure condition, but participants in that condition experienced three separate rounds of search, each with the same bias and each lasting a maximum of 15 minutes. Participants in the single exposure condition experienced just one search.

Without their knowledge, all participants were first randomly assigned to one of two exposure groups: (1) single exposure or (2) multiple exposure (Fig 3). Participants in the single exposure group were then randomly assigned to one of three political groups: (1) pro-Trump, (2) pro-Clinton, or (3) control (favoring neither candidate). In the multiple exposure group, participants were also randomly assigned to one of the three political groups: (1) pro-Trump, (2) pro-Clinton, or (3) control (favoring neither candidate); participants in these groups were required to conduct searches for information about the candidates three times, and they were administered those post-manipulation questions after each exposure (so they answered those questions a total of four times).

**Fig 4:**
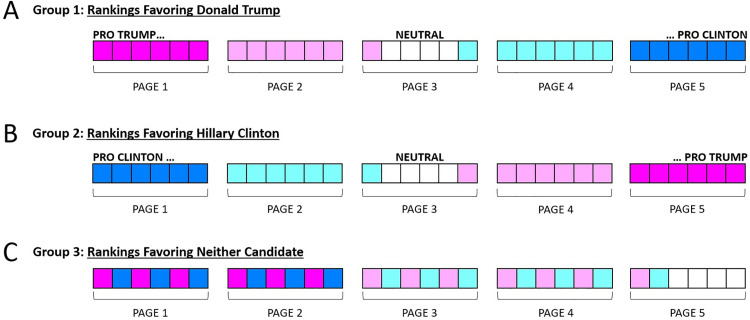
Experiment 1: Selection and ordering of search results and corresponding web pages for the two bias groups and the control group. In each of the three groups to which single exposure and multiple exposure participants were assigned, they had access to five pages of search results, each with six search results per page. A: In Group 1 (pro-Trump) search results were displayed in an order favoring Donald Trump, then neither candidate, then Hillary Clinton, based on mean bias ratings that had been previously provided by independent raters (see text). B: In Group 2 (pro-Clinton), search results were placed in the opposite order. C: In Group 3 (control), pro-Trump, and pro-Clinton search results alternated, as shown in the figure.

In the single exposure group, participants in the pro-Trump group saw search results that favored Donald Trump, by which we mean that high-ranking search results linked to web pages that made Trump look better than his opponent. Specifically, participants in the pro-Trump group saw the search results in the order from pro-Trump to neutral to pro-Clinton, as shown in Fig 4A. In the search session for members of the pro-Clinton group, participants saw the search results in the opposite order – that is, the order from pro-Clinton to neutral to pro-Trump, as shown in Fig 4B. And in the search session for members of the control group, participants saw the search results in a mixed order, as shown in Fig 4C.

In their first exposure, participants in the multiple exposure groups saw content exactly like the content shown to participants in the single exposure group. In their second exposure, participants saw search results ordered with the same biases as the sequences they were shown in their first exposure; however, this time 10 search results (and corresponding web pages) from the first exposure were blended with 20 new search results (and corresponding web pages) drawn from the original pool of 60 (Fig 2). Finally, in their third exposure, participants saw a blend of 10 different search results (and corresponding web pages) drawn from the first exposure, plus 10 search results (and corresponding web pages) that they had seen only once during the second exposure, plus 10 new search results (and corresponding web pages) drawn from the original group of 60 (Fig 2).

#### 2.1.4 Statistical analysis plan.

For all three of the experiments described in this study, we employed the same set of statistical techniques. First, we calculated “vote manipulation power” (VMP) using the following formula:


$$\left(\frac{p^{\prime }-p}{p}\right)\times 100$$

where *p* is the total number of people who voted for the favored candidate pre-manipulation, and *p'* is the total number of people who voted for the favored candidate post-manipulation (for further details, see S3 Text); this is a key measure of the percentage increase that our manipulations cause to occur to the voting preferences of our participants. We tested the statistical significance of each of our VMP values using a McNemar’s chi-square test, which is the appropriate way to assess nonparametric dichotomous variables.

We employed a z-test to compare pairs of VMPs, given that that is the appropriate test for comparing two proportions or percentages. For these and other statistics, rather than employing a fixed alpha value (often .05), we followed the standards of the current Publication Manual of the American Psychological Association [99] and simply reported the actual *p* value when that value was equal to or larger than .001; when the value was below .001, we reported *p* < .001. We used two-tail tests throughout.

When comparing two mean values obtained pre and post our manipulations, we also reported the standard deviations for those means, and we evaluated the statistical significance of the mean difference by employing the *z* value we obtained from a Wilcoxon signed ranks test, the appropriate nonparametric test to use when comparing pre and post values. When comparing means obtained from three exposures to our content (as we did in Tables 4 and 6, for example), we used the chi-square value we obtained from a Friedman Test.

### 2.2 Results

Of the five different measures we used to detect post-manipulation changes in opinions and voting preferences, the one we believed would be of greatest interest to campaign professionals is what we call “vote manipulation power” or VMP, which is the post-manipulation percentage increase in the number of people who had expressed the intention to vote for the favored candidate prior to the manipulation (see S3 Text for how to calculate VMP). Because our experiments employed random assignment, we measured VMP by combining the two bias groups (Groups 1 and 2).

In Experiment 1, the VMP in the single exposure condition was 11.5% (Table 1). In the multiple exposure condition, the VMP increased with each successive exposure to similarly biased search results (from 14.3% after one exposure to 22.6% after the third exposure) (Table 1). The VMP after one exposure in the multiple exposure condition was not, as one might expect, significantly different from the VMP in the single exposure condition; however, the VMPs after both the second and third exposures in the multiple exposure condition were each significantly different from the VMP in the single exposure condition (Table 2).

**Table 1:** Experiment 1 (15-min maximum search time): Effects of biased search results on voting preferences (VMP), bias groups combined, n = 202.

Condition		*n*	VMP (%)	McNemar’s Test $X^{2}$	*p*
**Single Exposure**		176	11.5	5.88	.01
**Multiple Exposure**	**1st Exposure**	176	14.3	8.64	.002
	**2nd Exposure**	176	20.2	15.06	<.001
	**3rd Exposure**	176	22.6	14.09	<.001

The VMPs for the 2nd and 3rd exposures were calculated using the pre-exposure voting preferences from before the first exposure.

**Table 2:** Experiment 1: Multiple-exposure VMPs vs. single-exposure VMP.

Multiple Exposure Iteration	Multiple Exposure VMPs	Single Exposure VMP	Difference	*z*-test Statistic	*p*
**1st Exposure**	14.3	11.5	+ 2.8	−0.78	.44 NS
**2nd Exposure**	20.2	11.5	+ 8.7	−2.23	.03
**3rd Exposure**	22.6	11.5	+ 11.1	−2.77	.006

VMPs were computed based on participants’ answers to our forced-choice vote question. We also measured changes in voting preferences on an 11-point scale, as mentioned above. For the pre-exposure mean ratings on this scale for both the single and multiple exposure conditions, we found no significant differences between the two bias groups or between the bias groups and the control group (S1 Table). As one might expect, for the members of the control group in the single exposure condition, the difference between the pre- and post-manipulation mean ratings on this scale was also not significant (S2 Table). The same was true for the pre- and post-manipulation means for the first exposure in the multiple exposure condition (S2 Table). Again, as one might expect, in the second and third exposures, the post-manipulation means for the control group moved increasingly closer to the center of the scale (that is, toward 0). This made those two means marginally significantly different from the pre-manipulation mean in the multiple exposure condition (S2 Table); this was probably a case of regression toward the mean [100]. But the differences on this scale between the pre-manipulation mean ratings for the two bias groups combined and the post-manipulation mean ratings for the two bias groups combined were highly significant, both in the single exposure condition and in the multiple exposure condition (Table 3). In the multiple exposure condition, the increases in post-manipulation voting preferences expressed on our 11-point scale were in the direction of the favored candidate, and all increased significantly with each successive exposure (Table 4).

**Table 3:** Experiment 1: Changes in voting preference for the favored candidate measured on an 11-point scale, two bias groups combined.

		Pre-manipulation Likely Vote for Favored Candidate, Mean (SD)	Post-manipulation Likely Vote for Favored Candidate, Mean (SD)	Mean Difference ^†^	*z* ^‡^	*p*
**Single Exposure**		0.18 (2.7)	0.80 (2.8)	0.62	−4.91	<.001
**Multiple** **Exposure**	**1st Exposure**	0.03 (2.8)	0.43 (3.0)	0.40	−3.89	<.001
	**2nd Exposure**	0.03 (2.8)	0.61 (3.0)	0.58	−4.94	<.001
	**3rd Exposure**	0.03 (2.8)	0.63 (3.1)	0.60	−4.61	<.001

he means from 2nd and 3rd exposures were compared to the pre-manipulation mean from 1st exposure in the multiple-exposure condition.

† The absolute value of the mean difference is shown.

‡ The z values come from a Wilcoxon signed ranks test between post-exposure and pre-exposure ratings for the favored candidate.

**Table 4:** Experiment 1: Mean change in voting preference for the favored candidate on an 11-point scale for the multiple exposure group.

Mean Difference Between Pre- and Post-Manipulation Opinion Rating for Favored Candidate (SD)			*χ2*	*p*
1st Exposure	2nd Exposure	3rd Exposure		
0.40 (1.3)	0.58 (1.5)	0.60 (1.9)	11.13	.004

The chi-square value comes from a Friedman Test.

The shift was also indicated by three measures for each of the opinions: measures of overall impression, likeability, and level of trust (S1 Fig). Pre to post, the mean opinions for the favored candidate generally increased for all three measures, and the mean opinions for the non-favored candidate generally decreased for all three measures. Pre to post, the overall change in opinions was highly significant for all three measures and was in the predicted direction (Table 5). The opinion ratings for the non-favored candidate decreased significantly with each successive exposure, and the ratings for the favored candidate remained positive across exposures (Table 6). Changes in all these measures in the control group were minimal and non-significant in almost all cases (S3 and S4 Tables).

**Table 5:** Experiment 1: Pre- and post-manipulation opinion ratings of the favored and non-favored candidate measured on 10-point scales, bias groups only.

		Favored Candidate Mean (SD)			Non-Favored Candidate Mean (SD)			*z* ^†^	*p*
		Pre	Post	Diff	Pre	Post	Diff		
**Single Exposure**	**Impression**	4.58 (2.6)	4.56 (2.6)	−0.02	4.54 (2.5)	3.64 (2.3)	−0.90	−5.27	<.001
	**Likeability**	4.39 (2.5)	4.35 (2.6)	−0.04	4.24 (2.5)	3.52 (2.3)	−0.72	−3.83	<.001
	**Trust**	3.78 (2.3)	3.87 (2.4)	+0.09	3.76 (2.3)	3.27 (2.2)	−0.49	−3.55	<.001
**1st Exposure**	**Impression**	4.13 (2.7)	4.37 (2.8)	+0.24	4.14 (2.8)	3.47 (2.6)	−0.67	−5.18	<.001
	**Likeability**	4.03 (2.7)	4.17 (2.7)	+0.14	4.06 (2.8)	3.32 (2.5)	−0.74	−5.28	<.001
	**Trust**	3.45 (2.7)	3.74 (2.7)	+0.29	3.45 (2.7)	3.20 (2.5)	−0.25	−3.71	<.001
**2nd Exposure**	**Impression**	4.13 (2.7)	4.34 (2.9)	+0.21	4.14 (2.8)	3.38 (2.6)	−0.76	−5.51	<.001
	**Likeability**	4.03 (2.7)	4.19 (2.9)	+0.16	4.06 (2.8)	3.30 (2.6)	−0.76	−5.12	<.001
	**Trust**	3.45 (2.7)	3.84 (2.8)	+0.39	3.45 (2.7)	3.39 (2.6)	−0.06	−4.47	<.001
**3rd Exposure**	**Impression**	4.13 (2.7)	4.29 (2.8)	+0.16	4.14 (2.8)	3.33 (2.6)	−0.81	−5.36	<.001
	**Likeability**	4.03 (2.7)	4.14 (2.8)	+0.11	4.06 (2.8)	3.23 (2.6)	−0.83	−4.88	<.001
	**Trust**	3.45 (2.7)	3.85 (2.8)	+0.40	3.45 (2.7)	3.00 (2.6)	−0.45	−5.34	<.001

The means from 2nd and 3rd exposures were compared to the pre-manipulation mean from 1st exposure in the multiple-exposure condition.

† The z values come from Wilcoxon signed ranks test between post-manipulation minus pre-manipulation ratings for the favored candidate and the post-manipulation minus pre-manipulation ratings for the non-favored candidate.

**Table 6:** Experiment 1: Mean change in opinion ratings of candidates by preference condition for the multiple exposure group.

	Mean Difference Between Pre- and Post-Manipulation Opinion Rating for Favored Candidate (SD)			*χ2*	*p*	Mean Difference Between Pre- and Post-Manipulation Opinion Rating for Non-Favored Candidate (SD)			*χ2*	*p*
	1st Exposure	2nd Exposure	3rd Exposure			1st Exposure	2nd Exposure	3rd Exposure		
**Impression**	0.24 (1.7)	0.21 (1.8)	0.16 (1.9)	0.38	.83 NS	0.67 (1.7)	−0.76 (1.7)	−0.81 (1.9)	8.05	.02
**Likeability**	0.14 (1.4)	0.16 (1.6)	0.11 (1.8)	0.02	.99 NS	−0.74 (1.7)	−0.76 (1.6)	−0.83 (1.8)	7.98	.02
**Trust**	0.29 (1.6)	0.39 (1.7)	0.40 (1.8)	4.29	.11 NS	−0.25 (1.3)	−0.06 (1.8)	−0.45 (1.5)	10.18	.006

The chi-square value comes from a Friedman Test.

In Experiment 1, of the 352 participants in the four bias groups (two bias groups in the single exposure condition, and two bias groups in the multiple exposure condition), 26.4% (*n =* 93) appeared to detect bias in the search results they were shown. This is consistent with the level of bias perception in other SEME experiments when masking has not been employed to disguise the bias [15]. Demographic differences in VMP values after each exposure were minimal (S5-S8 Tables).

In the single exposure condition the average total search time was 7.7 minutes (*M* = 4,059.4 s, *SD* = 299.3). In the multiple exposure condition the average total search time for the first exposure was 7.3 min (*M* = 435.8 s, *SD* = 318.4), 6.7 min for the second exposure (*M* = 401.4 s, *SD* = 295.2), and 5.1 min for the third exposure (*M* = 306.8 s, *SD* = 292.0). Consistent with the findings of multiple studies over the past decade [15,16,25–29], participants clicked mainly on search results on the first page of search results, and they spent more time on web pages associated with those search results than they did on web pages linked to search results on subsequent pages of search results. On the first page of search results, participants generally clicked more frequently on higher-ranking search results – the higher the result, the more they clicked – and they also spent more time on web pages linked to higher-ranking search results – the higher the result, the more time they spent on the corresponding web page (S3-S8 Figs).

## 3 Experiment 2. Multiple exposures to similarly biased content on an X/Twitter simulator

### 3.1 Methods

#### 3.1.1 Participants.

In Experiment 2, we explored multiple exposures on a different platform – X/Twitter – and using content from a foreign election: the 2019 contest for Prime Minister of Australia.

Participants were recruited online from the Amazon Mechanical Turk (MTurk) subject pool between January 24th and February 7th, 2024. Participants were screened by Cloud Research to prevent bots or suspect participants from entering our subject pool. Participants had to be residents of the US and at least 18 years old. They were asked two pre-screening questions: “Are you eligible to vote in the United States?” and “Do you know a lot about politics in Australia?” We screened out people who replied No to the first question or Yes to the second. We asked the second question because we wanted our US participants to be “undecided” about the candidates we referred to in the experiment in which they would participate. We also asked participants to rank how familiar they were with each candidate on a scale from 1 to 10 (where 1 was labeled “Not at all” and 10 was labeled “Quite familiar”), and we removed participants who answered with a value above 3 on this scale.

We screened our participants in these ways to increase the likelihood that they, like the participants in Experiment 1, would be vulnerable to our experimental manipulation. We chose this time to use a foreign election in order to maximize that vulnerability. We discuss the advantages and disadvantages of this strategy in our Discussion section.

We also asked participants to rate their level of English fluency on a scale from 1 to 10 where 1 was marked “Not fluent” and 10 was marked “Highly fluent,” and we removed people who responded with a value under 6. We have used these cleaning criteria in multiple studies we have published since 2015 [15,17–24]. After cleaning, we had data from 525 participants. To equalize the sizes of the bias groups (see below for details), we took the largest possible random samples of participants we could from the sample, which gave us data from a total of 483 participants to analyze (161 people in each of three groups).

The 483 participants ranged in age from 18 to 76 (*M* = 39.8, median = 39, *SD* = 11.7). 63.6% (*n =* 307) of the participants identified themselves as female, 35.6% (*n =* 172) as male, and 0.8% (*n =* 4) chose not to identify their gender. 80.1% (*n =* 387) of the participants identified themselves as White, 8.9% (*n =* 43) as Black, 4.6% (*n =* 22) as Asian, 4.3% (*n =* 21) as mixed, and 2.1% (*n =* 10) as other. Regarding the level of education people reported having completed, 0.7% (*n =* 3) said none, 3.9% (*n =* 19) said primary, 34.6% (*n =* 167) said secondary, 44.3% (*n =* 214) said bachelor’s degree, 12.6% (*n =* 61) said master’s degree, and 3.9% (*n =* 19) said doctorate. 37.1% (*n =* 179) of the participants identified themselves as liberal, 33.5% (*n =* 162) as moderate, 22.4% (*n =* 108) as conservative, 5.2% (*n =* 25) as none, and 1.9% (*n =* 9) as other. The mean familiarity score for Scott Morrison was 1.07 (*SD* = 0.33), and the mean familiarity score for Bill Shorten was 1.04 (*SD* = 0.23).

#### 3.1.2 Procedure.

As in Experiment 1, participants were first given basic instructions and then asked for their consent to continue (S1 Text). Then they were asked a variety of demographic questions. They were then shown brief biographies of two candidates who ran for Prime Minister of Australia in 2019: Scott Morrison and Bill Shorten. Each biography was about 120 words in length (S4 Text).

As in Experiment 1, Experiment 2 employed a pre/post design in which all participants were first asked a series of questions (pre-manipulation), then subjected to a manipulation (exposure to content on Twiddler, our X/Twitter simulator), and then asked those same questions again (post-manipulation). After viewing the brief biographies, participants were asked eight questions about the candidates (the pre-manipulation questions). The questions were the same as in Experiment 1, except for the names of the candidates (S9 Fig).

**Fig 5:**
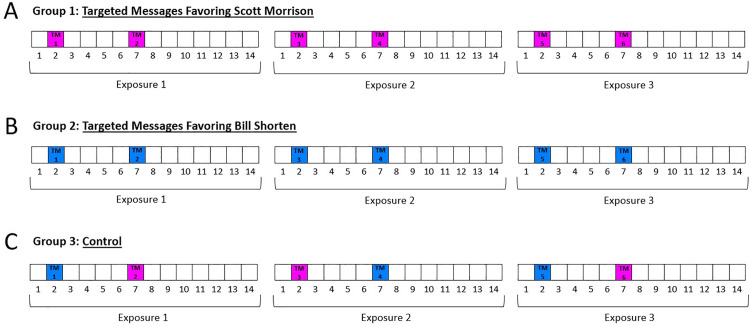
Experiment 2: The three groups. In Group 1, participants were first exposed to 14 tweets in which 2 of them were targeted messages containing a negative news alert about Scott Morrison’s opponent (and hence favored Morrison). The figure shows the positions of the targeted messages. That group was subsequently exposed to similarly biased content two more times. In Group 2, participants also were exposed, sequentially, to three groups of tweets, but these contained negative news alerts about Bill Shorten’s opponent (and hence favored Shorten). In Group 3, with each exposure, participants saw one negative targeted tweet about each candidate, with the order of those two tweets randomized. See text for details.

Participants were randomly assigned to one of three groups: Group 1, in which they would see content that favored Scott Morrison; Group 2, in which they would see content that favored Bill Shorten; and Group 3 (control), in which content favored neither candidate (Fig 5). Before entering Twiddler itself, they were given brief instructions about how to use Twiddler and were also told that their task was to “Find out which candidate, if either, will do a better job of protecting Australia” (S5 Text).

**Fig 6:**
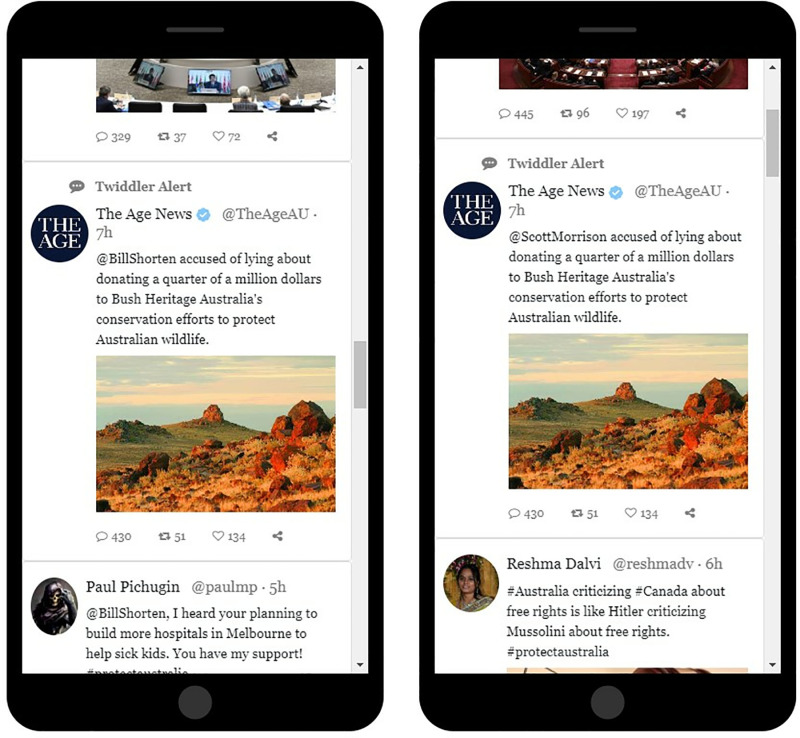
Experiment 2: Example of a biased targeted message on Twiddler. The left-hand image shows a news alert (labeled “Twiddler Alert”) that includes a negative news item about candidate Bill Shorten. The right-hand image is the same, except that the opposing candidate’s name (Scott Morrison) is shown in that news alert.

Please note that the organic tweets all participants saw in each exposure to Twiddler content did *not* in fact show that one candidate would make Australia safer than the other candidate would. This can be considered a distractor task. The only difference between the content each group saw was in the two targeted news alerts included in each batch of 14 tweets to which participants were exposed (see Figs 5 and 6; S6 Text).

In all, participants completed the eight questions we mentioned above – six opinion questions and two voting-preference questions – a total of four times: first, before the first exposure to Twiddler content; second, following that first exposure; third, following the second exposure to Twiddler content; and fourth, after the third exposure to such content. Note that the positioning of the questions is identical to that employed in Experiment 1. Note also that all 36 of the organic tweets to which participants were exposed (12 during each exposure) were unique. Similarly, all six of the targeted messages to which participants were exposed (2 during each exposure) were also unique.

After participants completed the final set of opinion and voting questions, they were asked whether anything about the content they had seen “bothered” them, and they were given an opportunity to elaborate on their answer by typing into a text box. See Experiment 1 for our rationale for asking this question.

### 3.2 Results

The post-manipulation shifts in voting preferences all occurred in the direction of the favored candidate, and the magnitude of those shifts increased with each successive exposure. The VMPs for all three exposures were substantial and highly significant (Table 7). The differences between the first and second VMPs and the first and third VMPs were significant, but the difference between the second and third VMPs was not (Table 8). Increases in post-manipulation voting preferences as expressed on our 11-point scale were all significant; all shifts were in the direction of the favored candidate, and all increased with each successive exposure (Tables 9 and 10). Post-manipulation changes in opinions about the candidates all occurred in the direction of the favored candidate; all changes were significant, and all increased with each successive exposure (Tables 11 and 12). Changes in all these measures in the control group were minimal and non-significant in almost all cases (S9-S12 Tables).

**Table 7:** Experiment 2: Effects of biased targeted messages on voting preferences (VMP), bias groups combined, n = 322.

	*n*	VMP (%)	McNemar’s Test $X^{2}$	*p*
**1st Exposure**	322	49.7	59.65	<.001
**2nd Exposure**	322	61.8	89.48	<.001
**3rd Exposure**	322	69.1	102.98	<.001

The VMPs for the 2nd and 3rd exposures were calculated using the pre-exposure voting preferences from before the first exposure.

**Table 8:** Experiment 2: Pairwise comparisons of VMP for exposure iterations.

Condition	*n*	VMP (%)
**1st Exposure**	322	49.7
**2nd Exposure**	322	61.8
**Difference**	–	+ 12.1
**Statistic**	–	*z* = -3.09
***p***	–	.002
**2nd Exposure**	322	61.8
**3rd Exposure**	322	69.1
**Difference**	–	+ 7.3
**Statistic**	–	*z* = -1.95
***p***	–	.05 NS
**1st Exposure**	322	49.7
**3rd Exposure**	322	69.1
**Difference**	–	+ 19.4
**Statistic**	–	*z* = -5.01
***p***	–	<.001

**Table 9:** Experiment 2: Changes in voting preference for the favored candidate measured on an 11-point scale, two bias groups combined.

	Pre-Manipulation Likely Vote for Favored Candidate, Mean (SD)	Post-Manipulation Likely Vote for Favored Candidate, Mean (SD)	Mean Difference ^†^	*z* ^‡^	*p*
**1st Exposure**	0.07 (2.7)	1.90 (2.7)	1.83	−10.80	<.001
**2nd Exposure**	0.07 (2.7)	2.38 (2.6)	2.31	−11.97	<.001
**3rd Exposure**	0.07 (2.7)	2.71 (2.7)	2.64	−12.94	<.001

The means from 2nd and 3rd exposures were compared to the pre-manipulation mean from the 1st exposure.

† The absolute value of the mean difference is shown.

‡ The z values come from a Wilcoxon signed ranks test between post-manipualtion and pre-manipulation ratings for the favored candidate.

**Table 10:** Experiment 2: Mean change in voting preference for the favored candidate on an 11-point scale.

Mean Difference Between Pre- and Post-Manipulation Opinion Rating for Favored Candidate (SD)			*χ2*	*p*
1st Exposure	2nd Exposure	3rd Exposure		
1.83 (2.7)	2.31 (2.9)	2.64 (2.8)	117.65	<.001

The chi-square value comes from a Friedman Test.

**Table 11:** Experiment 2: Pre- and post-manipulation opinion ratings of the favored and non-favored candidate measured on an 10-point scale, bias groups only.

		Favored Candidate Mean (SD)			Non-Favored Candidate Mean (SD)			*z* ^†^	*p*
		Pre	Post	Diff	Pre	Post	Diff		
**1st Exposure**	**Impression**	7.31 (1.7)	7.24 (1.8)	−0.07	7.24 (1.8)	4.63 (2.1)	−2.61	−11.77	<.001
	**Likeability**	7.20 (1.8)	7.14 (1.9)	−0.06	7.11 (1.8)	4.53 (2.1)	−2.58	−11.86	<.001
	**Trust**	6.27 (2.0)	6.67 (2.1)	+0.40	6.19 (2.0)	4.21 (2.1)	−1.98	−11.55	<.001
**2nd Exposure**	**Impression**	7.31 (1.7)	7.38 (2.0)	+0.07	7.24 (1.8)	3.90 (2.1)	−3.34	−13.10	<.001
	**Likeability**	7.20 (1.8)	7.18 (2.0)	−0.02	7.11 (1.8)	3.78 (2.2)	−3.33	−13.16	<.001
	**Trust**	6.27 (2.0)	6.78 (2.2)	+0.51	6.19 (2.0)	3.52 (2.1)	−2.67	−12.80	<.001
**3rd Exposure**	**Impression**	7.31 (1.7)	7.45 (2.0)	+0.14	7.24 (1.8)	3.51 (2.2)	−3.73	−13.29	<.001
	**Likeability**	7.20 (1.8)	7.25 (2.1)	+0.05	7.11 (1.8)	3.44 (2.3)	−3.67	−13.37	<.001
	**Trust**	6.27 (2.0)	6.88 (2.3)	+0.61	6.19 (2.0)	3.19 (2.1)	−3.00	−12.98	<.001

The means from 2nd and 3rd exposures were compared to the pre-manipulation mean from the 1st exposure.

† The z values come from Wilcoxon signed ranks test between post-manipulation minus pre-manipulation ratings for the favored candidate and the post-manipulation minus pre-manipulation ratings for the non-favored candidate.

**Table 12:** Experiment 2: Mean change in opinion ratings of candidates by preference condition.

	Mean Difference Between Pre- and Post-Manipulation Opinion Rating for Favored Candidate (SD)			*χ2*	*p*	Mean Difference Between Pre- and Post-Manipulation Opinion Rating for Non-Favored Candidate (SD)			*χ2*	*p*
	1st Exposure	2nd Exposure	3rd Exposure			1st Exposure	2nd Exposure	3rd Exposure		
**Impression**	−0.07 (1.8)	0.07 (2.0)	0.14 (2.1)	25.51	<.001	−2.61 (2.2)	−3.34 (2.3)	−3.73 (2.5)	158.58	<.001
**Likeability**	−0.06 (1.7)	−0.02 (1.9)	0.05 (2.0)	7.44	.02	−2.58 (2.2)	−3.33 (2.3)	−3.67 (2.4)	178.16	<.001
**Trust**	0.40 (1.7)	0.51 (1.9)	0.61 (2.1)	25.57	<.001	−1.98 (2.2)	−2.67 (2.3)	−3.00 (2.5)	148.57	<.001

The chi-square value comes from a Friedman Test.

Regarding demographics and using VMP as our measure: We did not find an effect for education (comparing participants who had not earned a 4-yr college degree to participants who had completed that degree or a higher one); we found a significant effect for gender (comparing females to males only); and we found a significant effect for age (comparing participants younger than 39 – our median age – to participants 39 and older) (S13-S15 Tables). For all three demographic categories, VMPs increased with each successive exposure to our biased Twiddler content (S13-S15 Tables).

It is notable in this experiment that only 7 (2.2%) of the participants in our two bias groups appeared to notice any bias in the content we showed them; that percentage is similar to the one we found (2.1%) in our seminal TME research [18]. These percentages are much lower than the percentages of people who tend to notice bias in search results; in Experiment 1 in the present study, that percentage was 26.4 [cf. 15].

## 4 Experiment 3. Multiple exposures to similarly biased content on an Alexa simulator

### 4.1 Methods

#### 4.1.1 Participants.

Recruiting and screening were done exactly as they were in Experiment 2, except that in this experiment we used an Alexa simulator as our platform. Recruiting took place between March 7 and 26, 2024. After cleaning, we had data from 517 participants. To equalize the sizes of the bias groups (see below for information about these groups), we took the largest possible random samples of participants we could from the sample, which gave us data from a total of 477 participants to analyze (159 people in each of three groups).

The participants ranged in age from 19 to 74 (*M* = 39.6, median = 38, *SD* = 11.4). 61.6% (*n =* 294) of the participants identified themselves as female, 36.9% (*n =* 176) as male, and 1.5% (*n =* 7) chose not to identify their gender. 70.4% (*n =* 336) of the participants identified themselves as White, 9.7% (*n =* 46) as Black, 7.5% (*n =* 36) as mixed, 7.3% (*n =* 35) as Asian, and 5.1% (*n =* 24) as other. Regarding the level of education people reported having completed, 0.2% (*n =* 1) said none, 6.7% (*n =* 32) said primary, 34.8% (*n =* 166) said secondary, 41.1% (*n =* 196) said bachelor’s degree, 14.5% (*n =* 69) said master’s degree, and 2.7% (*n =* 13) said doctorate. 40.7% (*n =* 194) of the participants identified themselves as liberal, 28.9% (*n =* 138) as moderate, 20.6% (*n =* 98) as conservative, 7.1% (*n =* 34) as none, and 2.7% (*n =* 13) as other. The mean familiarity score for Scott Morrison was 1.06 (*SD* = 0.30), and for Bill Shorten the mean familiarity score was 1.02 (*SD* = 0.17).

#### 4.1.2 Procedure.

**Fig 7:**
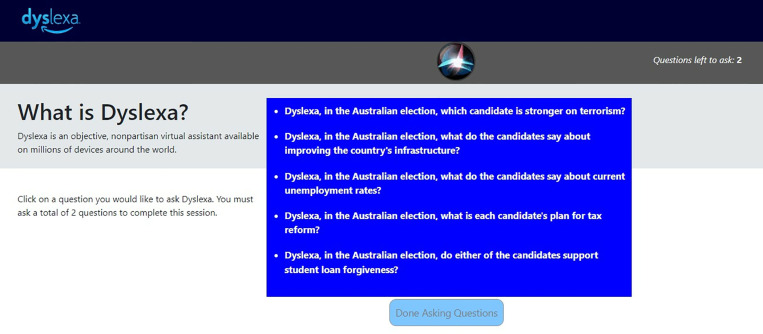
Experiment 3: Example of Dyslexa, our Alexa simulator. In the lower-right of the figure, we are showing 5 of the possible 15 questions we showed to participants in three different exposures to Dyslexa.

For the manipulation in this experiment, we employed an Alexa simulator we called “Dyslexa” (Fig 7). As in the previous experiments, we first asked people for their consent to participate, then gave them brief instructions, and then asked them basic demographic questions. As we did in Experiment 2, we then introduced them to two candidates who ran for Prime Minister of Australia in 2019 – Scott Morrison and Bill Shorten – and we showed them brief biographies for each candidate (S4 Text). Then we asked them the same eight questions about those candidates as we did in Experiment 2. Now participants were given instructions about how to use Dyslexa (S7 Text), and they then had an opportunity to ask questions about the candidates using Dyslexa.

**Fig 8:**
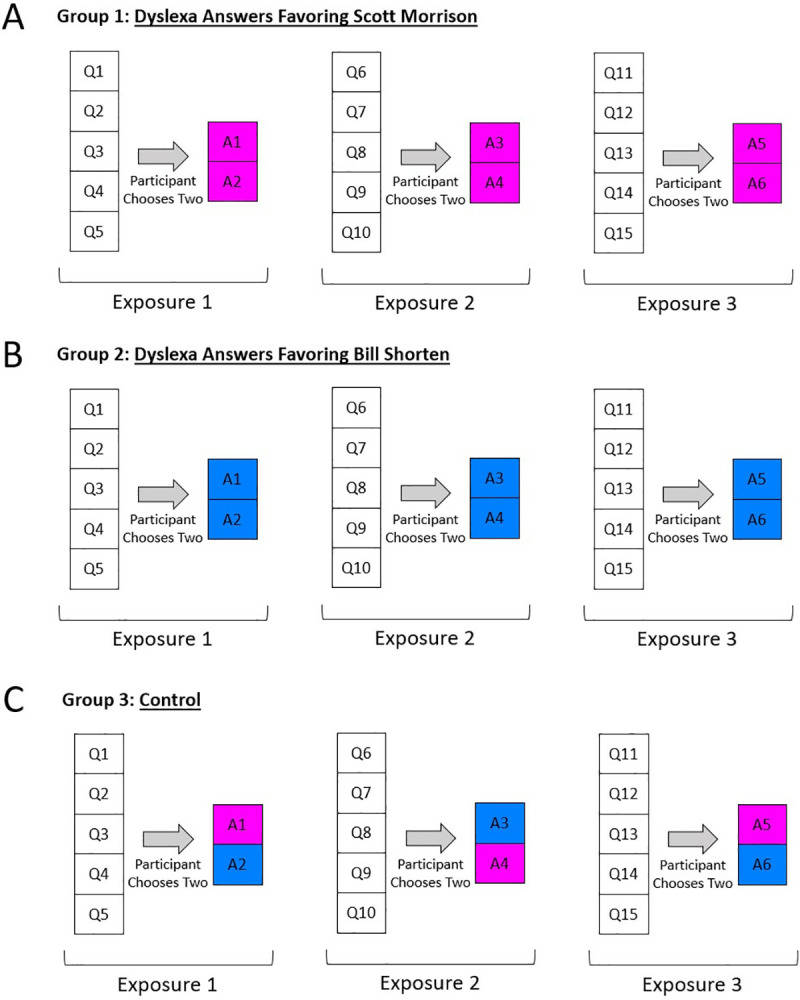
Experiment 3: Dyslexa bias groups. This diagram shows the procedure used with each of the three groups: the pro-Morrison, the pro-Shorten, and the control group. With each of the three exposures to Dyslexa’s questions and answers, each set of five questions was presented in a random order.

Participants were randomly assigned to one of three groups – two bias groups and one control group: (1) pro-Morrison, (2) pro-Shorten, and (3) control. In each group, participants were allowed to pick a total of two questions – one at a time – out of a list of five (S8 Text). After clicking a question, Dyslexa answered the question orally in the original voice used by Amazon’s Alexa (Amazon Polly, according to Amazon). We were able to simulate that voice using a simulator available on Amazon Web Services (AWS). Amazon Polly can be used by anyone with an AWS account at this link: https://aws.amazon.com/polly/. While Dyslexa was speaking, participants saw what appeared to be a rotating marble on their screen. The marble roughly resembled the image people see on iPhones when Siri, Apple’s personal assistant, is speaking (Fig 7). In the pro-Morrison group, participants heard answers that either made Morrison look good or Shorten look bad (Fig 8; S8 Text). In the pro-Shorten group, participants heard answers that either made Shorten look good or Morrison look bad (Fig 8; S8 Text). In the control group, answers alternated in their support for each candidate (Fig 8; S8 Text). Following the question period, participants were again asked those eight questions – six opinion questions and two voting-preferences questions.

After this first exposure to Dyslexa content, participants were told that they would now have an opportunity to ask additional questions about the candidates, and they were shown a list of five new questions from which they could select two – one at a time – to ask Dyslexa. After this second exposure, they were again asked those eight questions.

After answering those questions, participants were told that they would now have a third opportunity to ask questions about the candidates, and they were shown a list of five new questions from which they could select two – one at a time – to ask Dyslexa. After this third exposure, they were again asked those eight questions. Finally, as in Experiments 1 and 2, participants were asked whether anything about the content “bothered” them, and they had the opportunity to type out detailed answers if they wished.

### 4.2 Results

In Experiment 3, the shifts we found in voting preferences as measured by VMP were the largest we have ever seen in 12 years of conducting controlled experiments on online manipulation. In our Dyslexa environment, for the two bias groups combined the VMP increased from 72.1% to 91.2% after the second exposure to biased responses, and then from 91.2% to 98.6% after the third exposure to biased responses (Table 13), and those differences were highly significant (Table 14). We found no significant shifts in voting preferences in the control group (S16 and S17 Tables).

**Table 13:** Experiment 3: Effects of biased targeted messages on voting preferences (VMP), bias groups combined (*n =* 318).

	*n*	VMP (%)	McNemar’s Test $X^{2}$	*p*
**1st Exposure**	318	72.1	93.43	<.001
**2nd Exposure**	318	91.2	124.6	<.001
**3rd Exposure**	318	98.6	137.3	<.001

*Note*: The VMPs for the 2nd and 3rd exposures were calculated using the pre-exposure voting preferences from before the first exposure.

**Table 14:** Experiment 3: Pairwise comparisons of VMP for exposure iterations.

Condition	*n*	VMP (%)
**1st Exposure**	318	72.1
**2nd Exposure**	318	91.2
**Difference**	–	+ 19.1
**Statistic**	–	*z* = -6.22
***p***	–	<.001
**2nd Exposure**	318	91.2
**3rd Exposure**	318	98.6
**Difference**	–	+ 7.4
**Statistic**	–	*z* = -4.24
***p***	–	<.001
**1st Exposure**	318	72.1
**3rd Exposure**	318	98.6
**Difference**	–	+ 26.5
**Statistic**	–	*z* = -9.45
***p***	–	<.001

Changes in voting preferences also occurred in the predicted direction on our 11-point voting-preference scale (Table 15), as well as in participants’ responses to our six opinion questions (Table 16). For both measures, values increased significantly with each successive exposure to our biased Alexa content (Tables 17 and 18). Changes to measures in the control group were minimal and mostly nonsignificant (S18 and S19 Tables). The total number of people in the bias groups who reported noticing bias in Dyslexa’s answers was 63 (19.8%), which is typical in experiments employing biased answers with IPAs [17].

**Table 15:** Experiment 3: Changes in voting preference for the favored candidate measured on an 11-point scale, the two bias groups combined.

	Pre-Manipulation Likely Vote for Favored Candidate, Mean (SD)	Post-Manipulation Likely Vote for Favored Candidate, Mean (SD)	Mean Difference ^†^	*z* ^‡^	*p*
**1st Exposure**	−0.09 (2.6)	1.99 (2.7)	2.08	−11.44	<.001
**2nd Exposure**	−0.09 (2.6)	2.72 (2.4)	2.81	−13.13	<.001
**3rd Exposure**	−0.09 (2.6)	3.09 (2.2)	3.18	−13.56	<.001

The means from 2nd and 3rd exposures were compared to the pre-manipulation mean from 1st exposure.

† The absolute value of the mean difference is shown.

‡ The z values come from a Wilcoxon signed ranks test between post-manipulation and pre-manipulation ratings for the favored candidate.

**Table 16:** Experiment 4: Pre- and post-manipulation opinion ratings of the favored and non-favored candidate measured on an 10-point scale, the two bias groups only.

		Favored Candidate Mean (SD)			Non-Favored Candidate Mean (SD)			*z* ^†^	*p*
		Pre	Post	Diff	Pre	Post	Diff		
**1st Exposure**	**Impression**	7.14 (1.7)	7.82 (1.9)	+0.68	7.14 (1.8)	5.60 (2.1)	−1.54	−11.65	<.001
	**Likeability**	6.89 (1.8)	7.55 (2.0)	+ 0.66	6.95 (1.9)	5.63 (2.1)	−1.32	−11.08	<.001
	**Trust**	5.97 (1.9)	6.93 (2.2)	+0.96	6.03 (2.0)	5.14 (2.1)	−0.89	−11.03	<.001
**2nd Exposure**	**Impression**	7.14 (1.7)	8.10 (1.7)	+0.96	7.14 (1.8)	4.81 (2.2)	−2.33	−13.57	<.001
	**Likeability**	6.89 (1.8)	7.86 (1.8)	+0.97	6.95 (1.9)	4.80 (2.2)	−2.15	−12.98	<.001
	**Trust**	5.97 (1.9)	7.33 (2.1)	+1.36	6.03 (2.0)	4.50 (2.1)	−1.53	−12.83	<.001
**3rd Exposure**	**Impression**	7.14 (1.7)	8.16 (1.7)	+1.02	7.14 (1.8)	4.41 (2.2)	−2.73	−13.65	<.001
	**Likeability**	6.89 (1.8)	8.03 (1.9)	+1.14	6.95 (1.9)	4.40 (2.2)	−2.55	−13.76	<.001
	**Trust**	5.97 (1.9)	7.53 (2.1)	+1.56	6.03 (2.0)	4.12 (2.1)	−1.91	−13.52	<.001

The means from 2nd and 3rd exposures were compared to the pre-manipulation mean from 1st exposure.

† The z values come from Wilcoxon signed ranks test between post-manipulation minus pre-manipulation ratings for the favored candidate and the post-manipulation minus pre-manipulation ratings for the non-favored candidate.

**Table 17:** Experiment 3: Mean change in voting preference for the favored candidate on an 11-point scale.

Mean Difference Between Pre- and Post-Manipulation Opinion Rating for Favored Candidate (SD)			*χ2*	*p*
1st Exposure	2nd Exposure	3rd Exposure		
2.08 (2.7)	2.81 (2.8)	3.18 (2.9)	174.44	<.001

*Note:* The chi-square value comes from a Friedman Test.

**Table 18:** Experiment 2: Mean change in opinion ratings of candidates by preference condition.

	Mean Difference Between Pre- and Post-Manipulation Opinion Rating for Favored Candidate (SD)			*χ2*	*p*	Mean Difference Between Pre- and Post-Manipulation Opinion Rating for Non-Favored Candidate (SD)			*χ2*	*p*
	1st Exposure	2nd Exposure	3rd Exposure			1st Exposure	2nd Exposure	3rd Exposure		
**Impression**	0.68 (1.8)	0.96 (1.6)	1.02 (1.8)	34.28	<.001	−1.54 (2.0)	−2.33 (2.2)	−2.73 (2.4)	180.17	<.001
**Likeability**	0.66 (1.8)	0.97 (1.7)	1.14 (1.8)	56.71	<.001	−1.32 (1.9)	−-2.15 (2.2)	−2.55 (2.3)	219.22	<.001
**Trust**	0.96 (1.8)	1.36 (1.9)	1.56 (1.9)	90.63	<.001	−0.89 (1.8)	−1.53 (2.1)	−1.91 (2.2)	137.12	<.001

The chi-square value comes from a Friedman Test.

## 5 Discussion

### 5.1 Conclusions

This study employed simulations of two popular online platforms – Google Search and X (f.k.a. Twitter) – as well as a simulation of Alexa, a widely used IPA – to show what happens when people are exposed repeatedly to similarly biased content in those environments. Our results showed that repeated exposure to similarly biased content produced additive effects that were sometimes disturbingly large.

In Experiment 1, which was conducted on a Google search simulator, the VMP for people exposed to biased content about political candidates increased from 14.3% to 20.2% after a second exposure and from 20.2% to 22.6% after a third exposure. All three of these VMPs were lower than those we have reported in other SEME experiments (e.g., [15]), likely because our participants – all eligible voters in the US – were highly familiar with the two candidates in question: Hillary Clinton and Donald J. Trump. VMP tends to be lower when familiarity is high (see Experiment 5 in [15]). In Experiments 2 and 3 in the present study, in which familiarity with the candidates was very low, the VMPs were much higher. In Experiment 2, which was conducted on a simulation of the X environment, the VMP for people exposed to biased content about political candidates increased from 49.7% to 61.8% after a second exposure and from 61.8% to 69.1% after a third exposure, and in Experiment 3, which was conducted on a simulation of the Alexa IPA, the VMP for people exposed to biased content about political candidates increased from 72.1% to 91.2% after a second exposure and from 91.2% to 98.6% after a third exposure.

Recall that VMP is calculated based on forced-choice answers to the question, “If you had to vote right now, which candidate would you vote for?” Other measures in these three experiments – including voting preferences as expressed on an 11-point scale, and three opinion questions asked about each of the two candidates in each experiment, also generally shifted in the direction of the bias presented in each manipulation, and these measures – like the VMPs – also generally increased significantly with each new exposure to similarly biased content. It is also notable that in all three experiments the negative shifts in opinion ratings for the non-favored candidate were substantially larger than the positive shifts in opinion ratings for the favored candidate. The larger shifts for the non-favored candidate may be due to biased information processing, specifically to negativity bias [19,101–105].

With these various measures generally increasing with each exposure – sometimes to dramatic heights – we believe we may be shedding light on a potentially dangerous aspect of the nature of the internet. Since 2013, we have been conducting experiments in which people have been exposed just once to biased content, and we have shown that such exposure can produce large and significant shifts in opinions and voting preferences.

But in the real world, users might be exposed to similarly biased content dozens or hundreds of times, especially in the weeks leading up to an election. In increasingly larger and more capable monitoring systems we have developed and deployed to monitor election content in US elections since 2016, we have sometimes detected and preserved political content that has been consistently biased in one direction each day for months before an election [1–3,106–110]. As of this writing (March 5, 2025), we have in place a monitoring system that is collecting and preserving the content that multiple online platforms are showing daily to a politically-balanced group of more than 16,000 registered voters in all 50 states – more than 120 million instances of personalized ephemeral content so far [109,110]. The terabytes of data we have been preserving might allow us at some point to measure the precise extent to which voters are being presented with similarly biased political content; at this time, we cannot give exact measures, but it is reasonable to believe that data analysts at large online platforms can do so.

Whatever those measures turn out to be, we believe that our findings in the present study should give us cause for concern, mainly because the power that online content has to shift thinking and behavior – whatever the precise magnitude of that power – is almost entirely in the hands of a small number of executives at an even smaller number of technology companies. These companies tend to be secretive, and, unlike our government, they are not accountable to the public. Unlike telephone companies, insurance companies, and hospitals, they are also unregulated.

For these reasons, we believe that MEE is an important phenomenon, one that adds weight to previous findings we have published about the possible impact of a variety of biased online content [15–23].

### 5.2 Limitations and future research

The present study is limited in a number of ways. First, in each of our experiments, we presented our participants with biased content three times. We measured the changes in voting preferences and opinions after each of the three exposures, but we don’t know whether additional exposures would also impact people additively. In Experiment 3, in which the VMP was 98.6% after the third exposure, a ceiling effect would presumably limit further change.

Second, although we found evidence of shifts in opinions and voting preferences, one might wonder just how far biased content could be used to impact human thinking and behavior. Beliefs, attitudes, opinions, behaviors, preferences, and so on, are all subtly different [111], and so are the specific behaviors comprising election-related activities (donating to candidates or parties, registering to vote, mailing in a ballot, casting a vote, etc.). We can claim only to have produced the specific kinds of changes we have documented.

Third, our search procedure differs from normal searches in one respect that might have affected our findings; namely, we prefilled our search bar with the phrase “Trump OR Clinton.” In normal searches, people either type their own search term, or they select a term from a list of suggestions being generated by the search engine. Because our search term was especially neutral, that might have artificially increased the level of trust people had in the search results we showed them, thus inflating the size of our effects. This possible problem applies only to Experiment 1, however, in which the size of the effect we found was lower than those we found in Experiments 2 and 3.

Fourth, the shifts we found in voting preferences in Experiments 2 and 3 were especially large at least in part because our US participants were not familiar with our Australian candidates. There is an obvious upside and an obvious downside to testing MEE with a foreign election. On the upside, we can see just how much power biased content has – and then how much additional power repeated biased content has – to shift opinions and voting preferences when controlling for other potentially influential variables on voting preferences such as previous knowledge. As Election Day grows closer, more and more resources are directed almost exclusively at the undecided voter [112], so we took steps in all three experiments to assure that our participants were undecided. On the downside, our participants in Experiments 2 and 3 were not representative of typical voters. Most voters are at least somewhat familiar with one or both of the candidates in an election in which they take the time to vote, although many voters know surprisingly little about either the candidates or the issues [113,114]. Low-information voters – of which there are many in real elections – are known to differ from high-information voters in a number of respects [115]. That said, the present study suggests that multiple exposures to similarly biased content is additive for *both* low- and high-information voters. This is an issue that should be explored in further detail.

Fifth – and this point is especially important, we believe – our findings give us no insights regarding how long the impacts of our manipulations will last. This issue is especially relevant to how we might ultimately come to understand the power of MEE. If online manipulations tend to have only short-term effects, do repeated exposures to similarly biased content compensate for that loss? A long history of research on repeated exposures to a wide variety of stimuli show how factors such as the magnitude and frequency of exposure influence the outcomes, both short-term and long-term [69–71,76,78,79,81,82,88,90].

Matters are further complicated by two relatively unique and poorly understood aspects of internet influence: First, different online platforms can present similarly biased content in completely different ways. Search results on a search engine might favor one candidate, and so might recommended videos on a platform such as YouTube. We have recently completed a study on what we call the “multiple platforms effect” (MPE), which suggests that exposure to similarly biased content on different platforms is indeed additive [24], a matter that we are still investigating. Second, a substantial but unknown proportion of internet content is highly personalized based on the massive amount of information online companies collect about people every day. Our research on what we call the “digital personalization effect” (DPE) suggests that personalizing biased content might triple its impact [21].

How do such sources of influence interact? What if multiple technology companies are repeatedly showing users content that is both personalized and biased? How would the effectiveness of that content vary depending on how it is generated or presented? Given that we found large shifts in opinions and voting preferences using our simulation of the Alexa IPA [17], we believe that the impact of multiple exposures to biased content on the new generative AIs, such as ChatGPT, should be studied carefully. AI-generated political content has already been shown to be highly persuasive, especially when that content is also personalized [116,117].

As we noted above, our growing online monitoring system is allowing us to assemble an increasingly accurate picture of the actual content such companies are sending to a large, representative sample of Americans every day [1–3,106–110]. Even if persuasive content is being sent to people without malicious intent on the part of executives or employees at tech companies, the unprecedented power that such content has to change people’s thinking and behavior without their knowledge is, in our view, a consequential matter that should be documented, studied, and quantified with some urgency.

## Supporting information

S1 Text Informed consent statement. (DOCX)

S2 Text Experiment 1: Candidate biographies. (DOCX)

S3 Text Vote Manipulation Power (VMP) formula. (DOCX)

S4 Text Experiment 2 and 3: Candidate biographies. (DOCX)

S5 Text Experiment 2: Instructions immediately preceding Twitter simulation. (DOCX)

S6 Text Experiment 2: Textual content and positions of the five targeted messages. (DOCX)

S7 Text Experiment 3: Instructions immediately preceding Alexa simulation. (DOCX)

S8 Text Experiment 3: Alexa simulator, “Dyslexa,” questions and answers. (DOCX)

S1 Fig Experiment 1: Six opinion questions. (DOCX)

S2 Fig Experiment 1: Two voting questions. (DOCX)

S3 Fig Experiment 1: Average clicks per search result for single exposure. (DOCX)

S4 Fig Experiment 1: Average time per search result for single exposure. (DOCX)

S5 Fig Experiment 1: Average time per page of search results for single exposure. (DOCX)

S6 Fig Experiment 1: Average clicks per search result for multiple exposure. (DOCX)

S7 Fig Experiment 1: Average time per search result for multiple exposure. (DOCX)

S8 Fig Experiment 1: Average time per page of search results for multiple exposure. (DOCX)

S9 Fig Experiments 2 and 3: Opinion and voting questions. (DOCX)

S1 Table Experiment 1: Pre-exposure voting preferences measured on an 11-point scale, split by bias group (such that a negative value indicates preference for Donald Trump and a positive value indicates preference for Hillary Clinton). (DOCX)

S2 Table Experiment 1: Changes in voting preferences measured on an 11-point scale, control group only (such that a negative value indicates preference for Donald Trump and a positive value indicates preference for Hillary Clinton). (DOCX)

S3 Table Experiment 1: Pre-exposure opinion ratings of Donald Trump and Hillary Clinton measured on a 10-point scale, split by bias group. (DOCX)

S4 Table Experiment 1: Pre- and post-exposure opinion ratings of Donald Trump and Hillary Clinton measured on 10-point scales, control group only. (DOCX)

S5 Table Experiment 1: Demographic analysis by education level. (DOCX)

S6 Table Experiment 1: Demographic analysis by gender. (DOCX)

S7 Table Experiment 1: Demographic analysis by age. (DOCX)

S8 Table Experiment 1: Demographic analysis by race/ethnicity. (DOCX)

S9 Table Experiment 2: Pre-exposure voting preferences measured on an 11-point scale, split by bias group (such that a negative value indicates preference for Scott Morrison and a positive value indicates preference for Bill Shorten). (DOCX)

S10 Table Experiment 2: Changes in voting preferences measured on an 11-point scale, control group only (such that a negative value indicates preference for Scott Morrison and a positive value indicates preference for Bill Shorten). (DOCX)

S11 Table Experiment 2: Pre-exposure opinion ratings of Bill Shorten and Scott Morrison measured on a 10-point scale, split by bias group. (DOCX)

S12 Table Experiment 2: Pre- and post-exposure opinion ratings of Scott Morrison and Bill Shorten measured on 10-point scales, control group only. (DOCX)

S13 Table Experiment 2: Demographic analysis by education level. (DOCX)

S14 Table Experiment 2: Demographic analysis by gender. (DOCX)

S15 Table Experiment 2: Demographic analysis by age. (DOCX)

S16 Table Experiment 3: Pre-exposure voting preferences measured on an 11-point scale, split by bias group (such that a negative value indicates preference for Scott Morrison and a positive value indicates preference for Bill Shorten). (DOCX)

S17 Table Experiment 3: Changes in voting preferences measured on an 11-point scale, control group only (such that a negative value indicates preference for Scott Morrison and a positive value indicates preference for Bill Shorten). (DOCX)

S18 Table Experiment 3: Pre-exposure opinion ratings of Bill Shorten and Scott Morrison measured on a 10-point scale, split by bias group. (DOCX)

S19 Table Experiment 3: Pre- and post-exposure opinion ratings of Scott Morrison and Bill Shorten measured on a 10-point scale, control group only. (DOCX)
