# Supplementary material for: The “multiple exposure effect” (MEE): How multiple exposures to similarly biased online content can cause increasingly larger shifts in opinions and voting preferences
Source: PLoS One. 2025 May 12;20(5):e0322900. doi: 10.1371/journal.pone.0322900 (PMC12068600; doi:10.1371/journal.pone.0322900)
Supplement: S1 Text — (DOCX) [file pone.0322900.s001.docx]

**S1 Text: Informed consent statement.**

By clicking continue I understand that I must be 18 or over to participate in this study, that my participation is voluntary, that I am free to withdraw at any time, that I am providing information anonymously and that demographic information collected is confidential and cannot be used to identify me. I agree to allow the data collected to be used for future research projects, and I understand that completion and submission of this survey implies my consent to participate in the present study.
