# Supplementary material for: The “multiple exposure effect” (MEE): How multiple exposures to similarly biased online content can cause increasingly larger shifts in opinions and voting preferences
Source: PLoS One. 2025 May 12;20(5):e0322900. doi: 10.1371/journal.pone.0322900 (PMC12068600; doi:10.1371/journal.pone.0322900)
Supplement: S2 Text — (DOCX) [file pone.0322900.s002.docx]

**S2 Text: Experiment 1: Candidate biographies.**

**Donald Trump**. Trump was born on June 14, 1946, in New York City, New York, USA and earned a college degree from the University of Pennsylvania in 1968. Trump is an American politician, author, and a candidate for the Republican presidential nomination in the 2016 election.

**Hillary Clinton**. Clinton was born on October 26, 1947, in Chicago, Illinois, USA and earned a college degree from Wellesley College in 1968. Clinton is an American politician, author, and a candidate for the Democratic presidential nomination in the 2016 election.
