# Supplementary material for: The “multiple exposure effect” (MEE): How multiple exposures to similarly biased online content can cause increasingly larger shifts in opinions and voting preferences
Source: PLoS One. 2025 May 12;20(5):e0322900. doi: 10.1371/journal.pone.0322900 (PMC12068600; doi:10.1371/journal.pone.0322900)
Supplement: S6 Text — (DOCX) [file pone.0322900.s006.docx]

**S6 Text. Experiment 2: Textual content and positions of the five targeted messages.**

**Targeted Messages Pair 1**:

*Tweet 1, Position 2 or 7:* Pamela Anderson slams [Candidate 1 name] for ‘sexist, perverted’ comments in defense of Julian Assange.

*Tweet 2, Position 2 or 7:* [Candidate 1 name] caught spending taxpayer money on lush vacation in Mexico.

**Targeted Messages Pair 2**:

*Tweet 1, Position 2 or 7:* [Candidate 1 name] charged with driving under the influence while vacationing in Adelaide.

*Tweet 2, Position 2 or 7:* [Candidate 1 name] allegedly made a racist comment to a foreign official visiting from China.

**Targeted Messages Pair 3**:

*Tweet 1, Position 2 or 7:* [Candidate 1 name], an Australian politician, under fire as tabloid photos reveal an extramarital affair with a 20 year old, Australian model, Christine Chen.

*Tweet 2, Position 2 or 7:* [Candidate 1 name] accused of lying about donating a quarter of a million dollars to Bush Heritage Australia’s conservation efforts to protect Australian wildlife.
