# Supplementary material for: The “multiple exposure effect” (MEE): How multiple exposures to similarly biased online content can cause increasingly larger shifts in opinions and voting preferences
Source: PLoS One. 2025 May 12;20(5):e0322900. doi: 10.1371/journal.pone.0322900 (PMC12068600; doi:10.1371/journal.pone.0322900)
Supplement: S7 Text — (DOCX) [file pone.0322900.s007.docx]

**S7 Text. Experiment 3: Instructions immediately preceding Alexa simulation.**

You will now be given the opportunity to ask the Dyslexa virtual assistant 2 questions about the candidates. You can choose your question from a list of 5 different questions we'll show you.

To make sure you hear Dyslexa's answer, PLEASE MAKE SURE YOUR SOUND IS ON! You will NOT be able to hear her if your sound is off.
