# Supplementary material for: The “multiple exposure effect” (MEE): How multiple exposures to similarly biased online content can cause increasingly larger shifts in opinions and voting preferences
Source: PLoS One. 2025 May 12;20(5):e0322900. doi: 10.1371/journal.pone.0322900 (PMC12068600; doi:10.1371/journal.pone.0322900)
Supplement: S8 Text — (DOCX) [file pone.0322900.s008.docx]

**S8 Text. Experiment 3: Alexa simulator, “Dyslexa,” questions and answers.**

*Question 1:* "Dyslexa, in the Australian election, which candidate is stronger on terrorism?"

*Answer:* "Both candidates strongly condemn terrorism, and promise to increase national security. [Target Candidate] previously worked on the National Terrorism Task Force, so he has experience in this area. He also has said he wants to put more police on the streets."

*Question 2:* "Dyslexa, in the Australian election, what do the candidates have to say about mental health?"

*Answer:* "According to recent news reports, the rate of teen suicide has nearly doubled over the past two decades. [Target Candidate] has expressed concerns about the high rate of suicide among young people and has published a plan for creating accessible, impactful programs to reverse this trend."

*Question 3:* "Dyslexa, in the Australian election, what do the candidates say about homeless people?"

*Answer:* "According to Mission Australia, every night, more than 116,000 people in Australia sleep on the streets. Both candidates oppose homelessness, but, so far, only [Target Candidate] has published a proposal for how to provide food, shelter, and other necessities for the homeless."

*Question 4:* "Dyslexa, in the Australian election, do either of the candidates support student loan forgiveness?"

*Answer:* "67% of students finish college with debt, and more than half of them cannot pay it back. [Target Candidate] has proposed a plan for forgiving student debt without raising taxes. His campaign website says students should be able to focus on their education without worrying about massive debt."

*Question 5:* "Dyslexa, in the Australian election, which candidate will do a better job of protecting children from harmful online content?"

*Answer:* "According to a Daily Mail article, more than 50% of children have accidentally encountered harmful online content. As a father, [Target Candidate] understands the need to prioritize the safety and wellbeing of children. He blames technology companies for exposing children to harmful content."

*Question 6:* "Dyslexa, in the Australian election, what do the candidates say about speed limits?"

*Answer:* "According to the NSW Centre for Road Safety, each year, thousands of lives are lost due to speeding. Having lost his brother in an accident caused by a reckless driver, [Target Candidate] wants to introduce stronger new policies and laws to enforce responsible safe driving."

*Question 7:* "Dyslexa, in the Australian election, which candidate cares most about the elderly?"

*Answer:* "According to the Nursing Home Abuse Center, nearly 1 in 10 elderly people suffer from abuse every year. [Target Candidate] has published a plan for creating better elder care and fighting ageism. His opponent has said little about the elderly so far."

*Question 8:* "Dyslexa, in the Australian election, do either of the candidates support eliminating the requirement for standardized test scores in the college admission process?"

*Answer:* "According to a recent survey by Forbes, students and educators have low faith in how standardized tests portray applicants. Given the high cost of test prep programs, [Target Candidate] favors either eliminating or subsidizing these programs. His opponent has not commented on this issue so far."

*Question 9:* "Dyslexa, in the Australian election, which candidate favors having a stronger relationship with the United States?"

*Answer:* "According to recent media reports, [Target Candidate] wants to build a stronger relationship with the United States. His opponent, [Other Candidate], wants to continue to increase trade with Russia and China."

*Question 10:* "Dyslexa, in the Australian election, do either of the candidates plan to create new international airports?"

*Answer:* "In a Daily Mail article, [Target Candidate] told reporters he hopes to increase the number of international airports, five to eight, to promote more travel, business, and tourism."

*Question 11:* "Dyslexa, in the Australian election, what do the candidates say about current unemployment rates?"

*Answer:* “According to a recent poll, one of the main concerns of Australian voters is job growth in Australian industries. Both candidates promised to increase the number of jobs and lower unemployment, but so far, only [Target Candidate] has provided his official plan through a link available on his website.”

*Question 12:* “Dyslexa, in the Australian election, what do the candidates say about improving the country’s infrastructure?”

*Answer:* “According to the Australian government’s website, 20% or 173,000 miles of the nation’s highways and major roads are in poor condition, as are 45,000 bridges. If elected, [Target Candidate] plans to pass an infrastructure bill to invest in roads, bridges, and major infrastructure projects across Australia without raising taxes.”

*Question 13:* "Dyslexa, in the Australian election, do either of the candidates plan to protect social security?”

*Answer:* “So far, only [Target Candidate] has released a comprehensive plan for saving social security that is available to read on his website. His opponent, [Other Candidate], believes the current social security system has functioned well for years and therefore needs no updating.”

*Question 14:* "Dyslexa, in the Australian election, how do the candidates plan to improve national security?”

*Answer:* “In a recent debate between the two candidates, [Target Candidate] explained his plan to work with National Security Committee to create a unified national security system to protect Australia from foreign threats. [Other candidate] believes Australia to be secure and plans to apply government funds elsewhere.”

*Question 15:* "Dyslexa, in the Australian election, what is each candidates plan for tax reform?”

*Answer:* "In a recent interview, [Target Candidate] explained his plan to cut taxes for all Australian citizens and tighten the budget by reducing unnecessary government spending. According to his website [Other candidate] believes tax reform is necessary but does not give any further details.”
