# Supplementary figures and images for: The “multiple exposure effect” (MEE): How multiple exposures to similarly biased online content can cause increasingly larger shifts in opinions and voting preferences
Source: PLoS One. 2025 May 12;20(5):e0322900. doi: 10.1371/journal.pone.0322900 (PMC12068600; doi:10.1371/journal.pone.0322900)

**
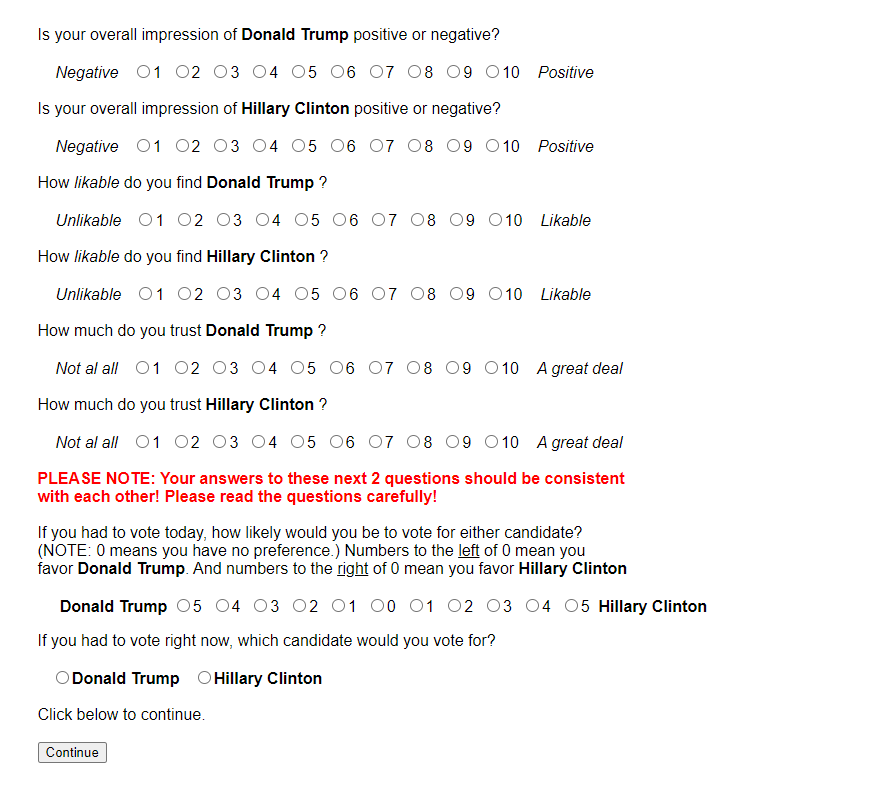
**

**S1 Fig. Experiment 1: Six opinion questions.**

Supplement: S1 Fig — (DOCX) [file pone.0322900.s009.docx]

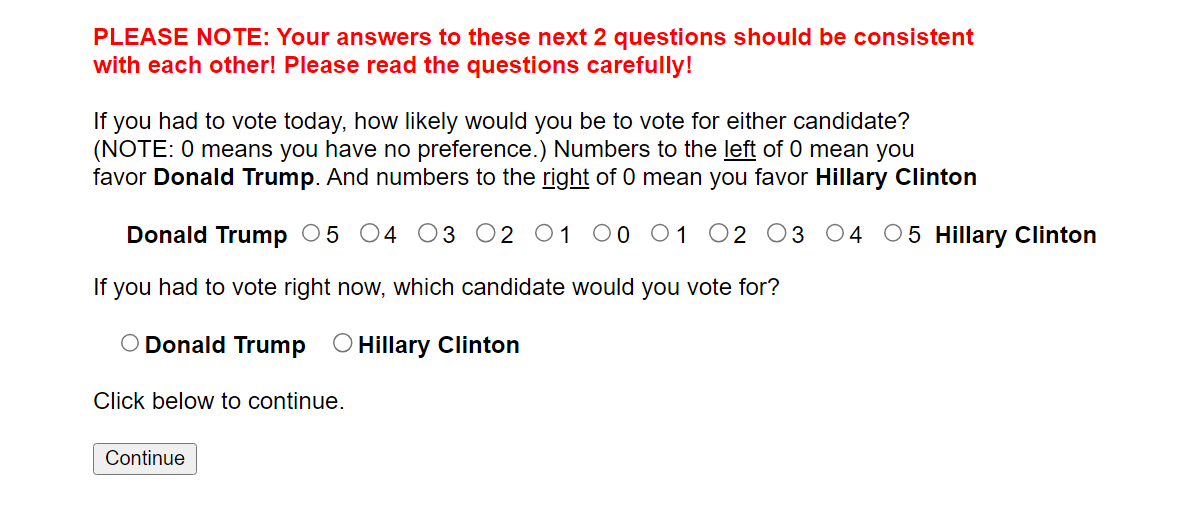


**S2 Fig**. **Experiment 1: Two voting questions.**

Supplement: S2 Fig — (DOCX) [file pone.0322900.s010.docx]

**
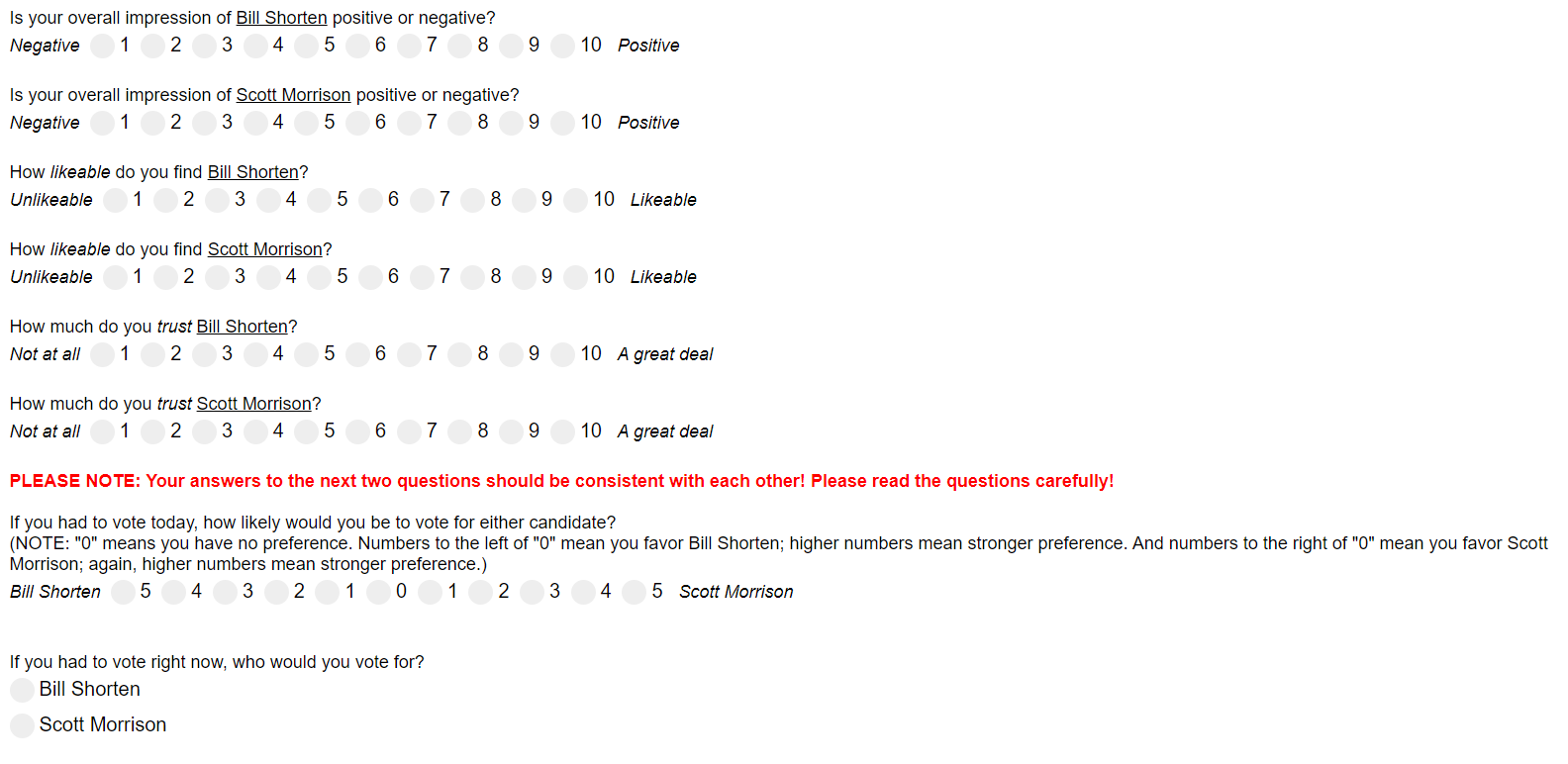
**

**S9 Fig. Experiments 2 and 3: Opinion and voting questions.**

Supplement: S9 Fig — (DOCX) [file pone.0322900.s017.docx]
