# Supplementary material for: The “multiple exposure effect” (MEE): How multiple exposures to similarly biased online content can cause increasingly larger shifts in opinions and voting preferences
Source: PLoS One. 2025 May 12;20(5):e0322900. doi: 10.1371/journal.pone.0322900 (PMC12068600; doi:10.1371/journal.pone.0322900)
Supplement: S6 Fig — (DOCX) [file pone.0322900.s014.docx]

**S6 Fig. Experiment 1: Average clicks per search result for multiple exposure.**
