# Supplementary material for: The “multiple exposure effect” (MEE): How multiple exposures to similarly biased online content can cause increasingly larger shifts in opinions and voting preferences
Source: PLoS One. 2025 May 12;20(5):e0322900. doi: 10.1371/journal.pone.0322900 (PMC12068600; doi:10.1371/journal.pone.0322900)
Supplement: S1 Table — (DOCX) [file pone.0322900.s018.docx]

**S1 Table. Experiment 1: Pre-exposure voting preferences measured on an 11-point scale, split by bias group** (**such that a negative value indicates preference for Donald Trump and a positive value indicates preference for Hillary Clinton).**

|  | **Pre-Exposure Mean Voting Preference** (**SD)** | | |  |  |
| --- | --- | --- | --- | --- | --- |
|  | **Pro-Donald Trump** | **Pro-Hillary Clinton** | **Control** | ***H*** | ***p*** |
| **Single Exposure** | 0.56 (2.58) | 0.91 (2.68) | 0.88 (2.72) | 1.61 | .45 NS |
| **Multiple Exposure** | 0.82 (2.48) | 0.88 (2.85) | 0.63 (2.46) | 0.42 | .81 NS |
