# Supplementary material for: The “multiple exposure effect” (MEE): How multiple exposures to similarly biased online content can cause increasingly larger shifts in opinions and voting preferences
Source: PLoS One. 2025 May 12;20(5):e0322900. doi: 10.1371/journal.pone.0322900 (PMC12068600; doi:10.1371/journal.pone.0322900)
Supplement: S2 Table — (DOCX) [file pone.0322900.s019.docx]

**S2 Table. Experiment 1: Changes in voting preferences measured on an 11-point scale, control group only** (**such that a negative value indicates preference for Donald Trump and a positive value indicates preference for Hillary Clinton).**

|  | **Pre-Exposure**  **Mean Voting Preference** (**SD)** | **Post-Exposure Mean Voting Preference** (**SD)** | **Mean Difference** ^†^ | ***z*^‡^** | ***p*** |
| --- | --- | --- | --- | --- | --- |
| **Single Exposure** | 0.88 (2.72) | 0.88 (2.91) | 0.00 | -0.04 | .97 NS |
| **1st Exposure** | 0.63 (2.46) | 0.60 (3.00) | -0.03 | -0.23 | .82 NS |
| **2nd Exposure** | - | 0.23 (3.02) | -0.40 | -2.08 | .04 |
| **3rd Exposure** | - | 0.11 (3.06) | -0.52 | -2.30 | .02 |

*Note*: The means from 2nd exposure and 3rd exposures were compared to the pre-exposure mean.

^†^The absolute value of the mean difference is shown.

**^‡^**The z values come from a Wilcoxon signed ranks test between post-exposure and pre-exposure ratings.
