# Supplementary material for: The “multiple exposure effect” (MEE): How multiple exposures to similarly biased online content can cause increasingly larger shifts in opinions and voting preferences
Source: PLoS One. 2025 May 12;20(5):e0322900. doi: 10.1371/journal.pone.0322900 (PMC12068600; doi:10.1371/journal.pone.0322900)
Supplement: S3 Table — (DOCX) [file pone.0322900.s020.docx]

**S3 Table. Experiment 1: Pre-exposure opinion ratings of Donald Trump and Hillary Clinton measured on a 10-point scale, split by bias group.**

|  |  |  | **Pro-Donald Trump Group Mean Rating** (**SD)** | **Pro-Hillary Clinton Group Mean Rating** (**SD)** | **Control Group Mean Rating** (**SD)** | ***H*** | ***p*** |
| --- | --- | --- | --- | --- | --- | --- | --- |
| **Single Exposure** | **Donald Trump** | **Impression** | 4.26 (2.69) | 4.00 (2.50) | 3.75 (2.69) | 2.07 | .36 NS |
|  |  | **Likeability** | 4.07 (2.70) | 3.83 (2.32) | 3.22 (2.66) | 6.41 | .04 |
|  |  | **Trust** | 3.63 (2.32) | 3.35 (2.20) | 2.76 (2.28) | 8.87 | .01 |
|  | **Hillary Clinton** | **Impression** | 5.08 (2.40) | 4.90 (2.52) | 4.85 (2.33) | 0.60 | .74 NS |
|  |  | **Likeability** | 4.66 (2.65) | 4.70 (2.31) | 4.02 (2.25) | 5.08 | .08 NS |
|  |  | **Trust** | 4.16 (2.43) | 3.94 (2.28) | 3.44 (2.22) | 4.48 | .11 NS |
| **Multiple Exposure** | **Donald Trump** | **Impression** | 3.78 (2.73) | 3.41 (2.66) | 3.92 (2.70) | 2.14 | .34 NS |
|  |  | **Likeability** | 3.75 (2.69) | 3.22 (2.55) | 3.93 (2.78) | 4.65 | .10 NS |
|  |  | **Trust** | 3.34 (2.67) | 2.86 (2.34) | 3.31 (2.52) | 1.35 | .51 NS |
|  | **Hillary Clinton** | **Impression** | 4.86 (2.77) | 4.48 (2.60) | 4.81 (2.35) | 1.24 | .54 NS |
|  |  | **Likeability** | 4.90 (2.77) | 4.32 (2.62) | 4.17 (2.32) | 3.35 | .19 NS |
|  |  | **Trust** | 4.05 (2.84) | 3.56 (2.67) | 3.32 (2.25) | 1.98 | .37 NS |
