# Supplementary material for: The “multiple exposure effect” (MEE): How multiple exposures to similarly biased online content can cause increasingly larger shifts in opinions and voting preferences
Source: PLoS One. 2025 May 12;20(5):e0322900. doi: 10.1371/journal.pone.0322900 (PMC12068600; doi:10.1371/journal.pone.0322900)
Supplement: S4 Table — (DOCX) [file pone.0322900.s021.docx]

**S4 Table. Experiment 1: Pre- and post-exposure opinion ratings of Donald Trump and Hillary Clinton measured on 10-point scales, control group only.**

|  |  | **Donald Trump Mean** (**SD)** | |  | **Hillary Clinton Mean** (**SD)** | |  |  |  |
| --- | --- | --- | --- | --- | --- | --- | --- | --- | --- |
|  |  | **Pre** | **Post** | **Diff** | **Pre** | **Post** | **Diff** | ***z***^†^ | ***p*** |
| **Single Exposure** | **Impression** | 3.75 (2.69) | 3.33 (2.78) | -0.42 | 4.85 (2.33) | 4.43 (2.30) | -0.42 | -0.15 | .88 NS |
|  | **Likeability** | 3.22 (2.66) | 3.10 (2.62) | -0.12 | 4.02 (2.25) | 4.03 (2.19) | 0.01 | -0.48 | .63 NS |
|  | **Trust** | 2.76 (2.28) | 2.86 (2.47) | 0.10 | 3.44 (2.22) | 3.61 (2.22) | 0.17 | -0.42 | .67 NS |
| **1st Exposure** | **Impression** | 3.92 (2.70) | 3.52 (2.63) | -0.04 | 4.81 (2.35) | 4.19 (2.40) | -0.62 | -1.31 | .19 NS |
|  | **Likeability** | 3.93 (2.78) | 3.50 (2.79) | -0.43 | 4.17 (2.32) | 3.90 (2.58) | -0.27 | -0.76 | .45 NS |
|  | **Trust** | 3.31 (2.52) | 3.05 (2.47) | -0.26 | 3.32 (2.25) | 3.43 (2.43) | 0.11 | -1.89 | .06 NS |
| **2nd Exposure** | **Impression** | - | 3.84 (2.89) | -0.08 | - | 3.76 (2.55) | -1.05 | -3.25 | < .001 |
|  | **Likeability** | - | 3.66 (3.06) | -0.27 | - | 3.61 (2.53) | -0.56 | -1.12 | .26 NS |
|  | **Trust** | - | 3.25 (2.82) | -0.06 | - | 3.17 (2.42) | -0.15 | -0.37 | .71 NS |
| **3rd Exposure** | **Impression** | - | 3.92 (2.95) | 0.00 | - | 3.78 (2.47) | -1.03 | -3.17 | .002 |
|  | **Likeability** | - | 3.69 (3.10) | -0.24 | - | 3.44 (2.41) | -0.73 | -1.51 | .13 NS |
|  | **Trust** | - | 3.38 (2.87) | 0.07 | - | 3.22 (2.54) | -0.10 | -0.42 | .68 NS |

*Note*: The means from 2nd exposure and 3rd exposures were compared to the pre-exposure mean.

^†^The z values come from Wilcoxon signed ranks test between post-exposure minus pre-exposure ratings for Scott Morrison and the post-exposure minus pre-exposure ratings for Bill Shorten.
