# Supplementary material for: The “multiple exposure effect” (MEE): How multiple exposures to similarly biased online content can cause increasingly larger shifts in opinions and voting preferences
Source: PLoS One. 2025 May 12;20(5):e0322900. doi: 10.1371/journal.pone.0322900 (PMC12068600; doi:10.1371/journal.pone.0322900)
Supplement: S5 Table — (DOCX) [file pone.0322900.s022.docx]

**S5 Table. Experiment 1: Demographic analysis by education level.**

| **Condition** | **Level** | ***N*** | **VMP** (**%)** |
| --- | --- | --- | --- |
| **Single Exposure** | **< Bachelors** | 93 | 7.5 |
|  | **≥ Bachelors** | 83 | 16.3 |
|  | **Difference** | - | - 8.8 |
|  | **Statistic** | *-* | z = - 1.82 |
|  | ***p*** | - | .07 NS |
| **Multiple Exposure** |  |  |  |
| **First Exposure** | **< Bachelors** | 85 | 18.4 |
|  | **≥ Bachelors** | 91 | 10.9 |
|  | **Difference** | - | + 7.5 |
|  | **Statistic** | - | z = 1.41 |
|  | ***p*** | - | .16 NS |
| **Second Exposure** | **< Bachelors** | 85 | 26.3 |
|  | **≥ Bachelors** | 91 | 15.2 |
|  | **Difference** | - | + 11.1 |
|  | **Statistic** | - | z = 1.82 |
|  | ***p*** | - | .07 NS |
| **Third Exposure** | **< Bachelors** | 85 | 31.6 |
|  | **≥ Bachelors** | 91 | 15.2 |
|  | **Difference** | - | + 16.4 |
|  | **Statistic** | *-* | z = 2.58 |
|  | ***p*** | - | .01 |
