# Supplementary material for: The “multiple exposure effect” (MEE): How multiple exposures to similarly biased online content can cause increasingly larger shifts in opinions and voting preferences
Source: PLoS One. 2025 May 12;20(5):e0322900. doi: 10.1371/journal.pone.0322900 (PMC12068600; doi:10.1371/journal.pone.0322900)
Supplement: S6 Table — (DOCX) [file pone.0322900.s023.docx]

**S6 Table. Experiment 1: Demographic analysis by gender.**

| **Condition** |  | ***N*** | **VMP** (**%)** |
| --- | --- | --- | --- |
| **Single Exposure** | **Female** | 91 | 12.5 |
|  | **Male** | 85 | 10.4 |
|  | **Difference** | - | - 2.1 |
|  | **Statistic** | *-* | z = 0.44 |
|  | ***p*** | - | .66 NS |
| **Multiple Exposure** |  |  |  |
| **First Exposure** | **Female** | 92 | 13.7 |
|  | **Male** | 84 | 15.2 |
|  | **Difference** | - | + 1.5 |
|  | **Statistic** | - | z = - 0.28 |
|  | ***p*** | - | .78 NS |
| **Second Exposure** | **Female** | 92 | 21.6 |
|  | **Male** | 84 | 18.2 |
|  | **Difference** | - | - 3.4 |
|  | **Statistic** | - | z = 0.56 |
|  | ***p*** | - | .58 NS |
| **Third Exposure** | **Female** | 92 | 21.6 |
|  | **Male** | 84 | 24.2 |
|  | **Difference** | - | + 2.6 |
|  | **Statistic** | *-* | z = - 0.41 |
|  | ***p*** | - | .68 NS |
