# Supplementary material for: The “multiple exposure effect” (MEE): How multiple exposures to similarly biased online content can cause increasingly larger shifts in opinions and voting preferences
Source: PLoS One. 2025 May 12;20(5):e0322900. doi: 10.1371/journal.pone.0322900 (PMC12068600; doi:10.1371/journal.pone.0322900)
Supplement: S7 Table — (DOCX) [file pone.0322900.s024.docx]

**S7 Table. Experiment 1: Demographic analysis by age.**

| **Condition** |  | ***N*** | **VMP** (**%)** |
| --- | --- | --- | --- |
| **Single Exposure** | **< 30** | 69 | 15.3 |
|  | **≥ 30** | 107 | 8.8 |
|  | **Difference** | - | - 6.5 |
|  | **Statistic** | *-* | z = 1.33 |
|  | ***p*** | - | .18 NS |
| **Multiple Exposure** |  |  |  |
| **First Exposure** | **< 30** | 83 | 20.0 |
|  | **≥ 30** | 93 | 10.2 |
|  | **Difference** | - | - 9.8 |
|  | **Statistic** | - | z = 1.83 |
|  | ***p*** | - | .07 NS |
| **Second Exposure** | **< 30** | 83 | 20.0 |
|  | **≥ 30** | 93 | 20.4 |
|  | **Difference** | - | + 0.4 |
|  | **Statistic** | - | z = - 0.07 |
|  | ***p*** | - | .94 NS |
| **Third Exposure** | **< 30** | 83 | 22.9 |
|  | **≥ 30** | 93 | 22.4 |
|  | **Difference** | - | - 0.5 |
|  | **Statistic** | *-* | z = 0.08 |
|  | ***p*** | - | .94 NS |
