# Supplementary material for: The “multiple exposure effect” (MEE): How multiple exposures to similarly biased online content can cause increasingly larger shifts in opinions and voting preferences
Source: PLoS One. 2025 May 12;20(5):e0322900. doi: 10.1371/journal.pone.0322900 (PMC12068600; doi:10.1371/journal.pone.0322900)
Supplement: S8 Table — (DOCX) [file pone.0322900.s025.docx]

**S8 Table. Experiment 1: Demographic analysis by race/ethnicity.**

| **Condition** |  | ***N*** | **VMP** (**%)** |
| --- | --- | --- | --- |
| **Single Exposure** | **White** | 132 | 16.4 |
|  | **Non-White** | 42 | 0 |
|  | **Difference** | - | - 16.4 |
|  | **Statistic** | *-* | z = 2.80 |
|  | ***p*** | - | .01 |
| **Multiple Exposure** |  |  |  |
| **First Exposure** | **White** | 133 | 13.0 |
|  | **Non-White** | 43 | 20.0 |
|  | **Difference** | - | + 7.0 |
|  | **Statistic** | - | z = - 1.13 |
|  | ***p*** | - | .26 NS |
| **Second Exposure** | **White** | 133 | 14.5 |
|  | **Non-White** | 43 | 46.6 |
|  | **Difference** | - | + 32.1 |
|  | **Statistic** | - | z = -4.39 |
|  | ***p*** | - | < .001 |
| **Third Exposure** | **White** | 133 | 14.5 |
|  | **Non-White** | 43 | 60.0 |
|  | **Difference** | - | + 45.5 |
|  | **Statistic** | *-* | z = -5.94 |
|  | ***p*** | - | < .001 |
