# Supplementary material for: The “multiple exposure effect” (MEE): How multiple exposures to similarly biased online content can cause increasingly larger shifts in opinions and voting preferences
Source: PLoS One. 2025 May 12;20(5):e0322900. doi: 10.1371/journal.pone.0322900 (PMC12068600; doi:10.1371/journal.pone.0322900)
Supplement: S9 Table — (DOCX) [file pone.0322900.s026.docx]

**S9 Table. Experiment 2: Pre-exposure voting preferences measured on an 11-point scale, split by bias group** (**such that a negative value indicates preference for Scott Morrison and a positive value indicates preference for Bill Shorten).**

|  | **Pro-Scott Morrison** | **Pro-Bill Shorten** | **Control** | ***H*** | ***p*** |
| --- | --- | --- | --- | --- | --- |
| **Pre-Exposure Mean Voting Preference** (**SD)** | -0.07 (2.70) | 0.07 (2.61) | -0.19 (2.71) | 0.91 | .63 NS |
