# Supplementary material for: The “multiple exposure effect” (MEE): How multiple exposures to similarly biased online content can cause increasingly larger shifts in opinions and voting preferences
Source: PLoS One. 2025 May 12;20(5):e0322900. doi: 10.1371/journal.pone.0322900 (PMC12068600; doi:10.1371/journal.pone.0322900)
Supplement: S10 Table — (DOCX) [file pone.0322900.s027.docx]

**S10 Table. Experiment 2: Changes in voting preferences measured on an 11-point scale, control group only** (**such that a negative value indicates preference for Scott Morrison and a positive value indicates preference for Bill Shorten).**

|  | **Pre-Exposure**  **Mean Voting Preference** (**SD)** | **Post-Exposure Mean Voting Preference** (**SD)** | **Mean Difference** ^†^ | ***z*^‡^** | ***p*** |
| --- | --- | --- | --- | --- | --- |
| **1st Exposure** | -0.19 (2.71) | 0.14 (2.76) | 0.33 | -2.01 | .04 |
| **2nd Exposure** | - | -0.35 (2.70) | -0.16 | -0.75 | .46 NS |
| **3rd Exposure** | - | -0.37 (2.75) | -0.18 | -0.69 | .49 NS |

*Note*: The means from 2nd exposure and 3rd exposures were compared to the pre-exposure mean.

^†^The absolute value of the mean difference is shown.

**^‡^**The z values come from a Wilcoxon signed ranks test between post-exposure and pre-exposure mean ratings.
