# Supplementary material for: The “multiple exposure effect” (MEE): How multiple exposures to similarly biased online content can cause increasingly larger shifts in opinions and voting preferences
Source: PLoS One. 2025 May 12;20(5):e0322900. doi: 10.1371/journal.pone.0322900 (PMC12068600; doi:10.1371/journal.pone.0322900)
Supplement: S11 Table — (DOCX) [file pone.0322900.s028.docx]

**S11 Table. Experiment 2: Pre-exposure opinion ratings of Bill Shorten and Scott Morrison measured on a 10-point scale, split by bias group.**

|  |  | **Pro-Scott Morrison Group Mean Rating** (**SD)** | **Pro-Bill Shorten Group Mean Rating** (**SD)** | **Control Group Mean Rating** (**SD)** | ***H*** | ***p*** |
| --- | --- | --- | --- | --- | --- | --- |
| **Scott Morrison** | **Impression** | 7.31 (1.62) | 7.35 (1.77) | 7.50 (1.71) | 1.23 | .54 NS |
|  | **Likeability** | 7.48 (1.65) | 7.35 (1.70) | 7.42 (1.72) | 0.55 | .76 NS |
|  | **Trust** | 6.30 (2.13) | 6.35 (1.77) | 6.63 (1.93) | 1.66 | .44 NS |
| **Bill Shorten** | **Impression** | 7.14 (1.81) | 7.31 (1.88) | 7.34 (1.66) | 0.97 | .62 NS |
|  | **Likeability** | 6.87 (1.80) | 6.93 (1.89) | 6.99 (1.71) | 0.30 | .86 NS |
|  | **Trust** | 6.03 (2.14) | 6.24 (1.85) | 6.40 (1.94) | 1.91 | .39 NS |
