# Supplementary material for: The “multiple exposure effect” (MEE): How multiple exposures to similarly biased online content can cause increasingly larger shifts in opinions and voting preferences
Source: PLoS One. 2025 May 12;20(5):e0322900. doi: 10.1371/journal.pone.0322900 (PMC12068600; doi:10.1371/journal.pone.0322900)
Supplement: S12 Table — (DOCX) [file pone.0322900.s029.docx]

**S12 Table. Experiment 2: Pre- and post-exposure opinion ratings of Scott Morrison and Bill Shorten measured on 10-point scales, control group only.**

|  | |  | **Scott Morrison Mean** (**SD)** |  | | **Bill Shorten Mean** (**SD)** |  | |  |  |
| --- | --- | --- | --- | --- | --- | --- | --- | --- | --- | --- |
|  |  | | **Pre** | **Post** | **Diff** | **Pre** | **Post** | **Diff** | ***z***^†^ | ***p*** |
| **1st Exposure** | **Impression** | | 7.50 (1.71) | 7.26 (1.75) | -0.24 | 7.34 (1.66) | 7.10 (1.78) | -0.24 | -0.02 | .98 NS |
|  | **Likeability** | | 7.42 (1.72) | 7.03 (1.85) | -0.39 | 6.99 (1.71) | 6.90 (1.86) | -0.09 | -2.04 | .04 |
|  | **Trust** | | 6.63 (1.93) | 6.57 (1.96) | -0.06 | 6.40 (1.94) | 6.57 (1.97) | -0.17 | -1.27 | .20 NS |
| **2nd Exposure** | **Impression** | | - | 7.34 (1.79) | -0.16 | - | 6.98 (1.88) | -0.36 | -1.25 | .21 NS |
|  | **Likeability** | | - | 7.06 (1.76) | -0.36 | - | 6.65 (1.85) | -0.34 | -0.06 | .95 NS |
|  | **Trust** | | - | 6.71 (1.91) | -0.08 | - | 6.42 (2.01) | -0.02 | -0.80 | .42 NS |
| **3rd Exposure** | **Impression** | | - | 7.32 (1.85) | -0.18 | - | 7.01 (1.98) | -0.33 | -0.88 | .38 NS |
|  | **Likeability** | | - | 7.16 (1.80) | -0.26 | - | 6.85 (1.89) | -0.14 | -0.81 | .42 NS |
|  | **Trust** | | - | 6.81 (2.00) | 0.18 | - | 6.55 (2.05) | 0.15 | -0.31 | .76 NS |

*Note*: The means from 2nd exposure and 3rd exposures were compared to the pre-exposure mean.

^†^The z values come from Wilcoxon signed ranks test between post-exposure minus pre-exposure ratings for Scott Morrison and the post-exposure minus pre-exposure ratings for Bill Shorten.
