# Supplementary material for: The “multiple exposure effect” (MEE): How multiple exposures to similarly biased online content can cause increasingly larger shifts in opinions and voting preferences
Source: PLoS One. 2025 May 12;20(5):e0322900. doi: 10.1371/journal.pone.0322900 (PMC12068600; doi:10.1371/journal.pone.0322900)
Supplement: S13 Table — (DOCX) [file pone.0322900.s030.docx]

**S13 Table. Experiment 2: Demographic analysis by education level.**

| **Exposure Iteration** | **Level** | ***N*** | **VMP** (**%)** |
| --- | --- | --- | --- |
| **First Exposure** | **< Bachelors** | 125 | 48.3 |
|  | **≥ Bachelors** | 197 | 50.5 |
|  | **Difference** | - | + 2.2 |
|  | **Statistic** | - | *z* = - 0.38 |
|  | ***p*** | - | .70 NS |
| **Second Exposure** | **< Bachelors** | 125 | 66.7 |
|  | **≥ Bachelors** | 197 | 59.0 |
|  | **Difference** | - | - 7.7 |
|  | **Statistic** | - | *z* = 1.39 |
|  | ***p*** | - | .16 NS |
| **Third Exposure** | **< Bachelors** | 125 | 76.7 |
|  | **≥ Bachelors** | 197 | 64.8 |
|  | **Difference** | - | - 11.9 |
|  | **Statistic** | - | *z* = 2.26 |
|  | ***p*** | - | .02 |
