# Supplementary material for: The “multiple exposure effect” (MEE): How multiple exposures to similarly biased online content can cause increasingly larger shifts in opinions and voting preferences
Source: PLoS One. 2025 May 12;20(5):e0322900. doi: 10.1371/journal.pone.0322900 (PMC12068600; doi:10.1371/journal.pone.0322900)
Supplement: S14 Table — (DOCX) [file pone.0322900.s031.docx]

**S14 Table. Experiment 2: Demographic analysis by gender.**

| **Exposure Iteration** |  | ***N*** | **VMP** (**%)** |
| --- | --- | --- | --- |
| **First Exposure** | **Female** | 199 | 71.3 |
|  | **Male** | 119 | 22.4 |
|  | **Difference** | - | - 48.9 |
|  | **Statistic** | - | *z* = 8.45 |
|  | ***p*** | - | < .001 |
| **Second Exposure** | **Female** | 199 | 81.9 |
|  | **Male** | 119 | 37.3 |
|  | **Difference** | - | - 44.6 |
|  | **Statistic** | - | *z* = 8.08 |
|  | ***p*** | - | < .001 |
| **Third Exposure** | **Female** | 199 | 90.4 |
|  | **Male** | 119 | 43.3 |
|  | **Difference** | - | - 47.1 |
|  | **Statistic** | - | *z* = 9.13 |
|  | ***p*** | - | < .001 |
