# Supplementary material for: The “multiple exposure effect” (MEE): How multiple exposures to similarly biased online content can cause increasingly larger shifts in opinions and voting preferences
Source: PLoS One. 2025 May 12;20(5):e0322900. doi: 10.1371/journal.pone.0322900 (PMC12068600; doi:10.1371/journal.pone.0322900)
Supplement: S15 Table — (DOCX) [file pone.0322900.s032.docx]

**S15 Table. Experiment 2: Demographic analysis by age.**

| **Exposure Iteration** |  | ***N*** | **VMP** (**%)** |
| --- | --- | --- | --- |
| **First Exposure** | **< 39** | 164 | 58.1 |
|  | **≥ 39** | 158 | 40.5 |
|  | **Difference** | - | - 17.6 |
|  | **Statistic** | - | *z* = 3.16 |
|  | ***p*** | - | .002 |
| **Second Exposure** | **< 39** | 164 | 67.4 |
|  | **≥ 39** | 158 | 55.7 |
|  | **Difference** | - | - 11.7 |
|  | **Statistic** | - | *z* = 2.16 |
|  | ***p*** | - | .03 |
| **Third Exposure** | **< 39** | 164 | 74.4 |
|  | **≥ 39** | 158 | 63.3 |
|  | **Difference** | - | - 11.1 |
|  | **Statistic** | - | *z* = 2.15 |
|  | ***p*** | - | .03 |
