# Supplementary material for: The “multiple exposure effect” (MEE): How multiple exposures to similarly biased online content can cause increasingly larger shifts in opinions and voting preferences
Source: PLoS One. 2025 May 12;20(5):e0322900. doi: 10.1371/journal.pone.0322900 (PMC12068600; doi:10.1371/journal.pone.0322900)
Supplement: S16 Table — (DOCX) [file pone.0322900.s033.docx]

**S16** **Table. Experiment 3: Pre-exposure voting preferences measured on an 11-point scale, split by bias group** (**such that a negative value indicates preference for Scott Morrison and a positive value indicates preference for Bill Shorten).**

|  | **Pro-Scott Morrison** | **Pro-Bill Shorten** | **Control** | ***H*** | ***p*** |
| --- | --- | --- | --- | --- | --- |
| **Pre-Exposure Mean Voting Preference** (**SD)** | 0.11 (2.60) | -0.07 (2.69) | 0.21 (2.52) | 1.58 | .45 NS |
