# Supplementary material for: The “multiple exposure effect” (MEE): How multiple exposures to similarly biased online content can cause increasingly larger shifts in opinions and voting preferences
Source: PLoS One. 2025 May 12;20(5):e0322900. doi: 10.1371/journal.pone.0322900 (PMC12068600; doi:10.1371/journal.pone.0322900)
Supplement: S18 Table — (DOCX) [file pone.0322900.s035.docx]

**S18 Table. Experiment 3: Pre-exposure opinion ratings of Bill Shorten and Scott Morrison measured on a 10-point scale, split by bias group.**

|  |  | **Pro-Scott Morrison Group Mean Rating** (**SD)** | **Pro-Bill Shorten Group Mean Rating** (**SD)** | **Control Group Mean Rating** (**SD)** | ***H*** | ***p*** |
| --- | --- | --- | --- | --- | --- | --- |
| **Scott Morrison** | **Impression** | 7.14 (1.70) | 7.20 (1.83) | 7.13 (1.68) | 0.28 | .87 NS |
|  | **Likeability** | 6.95 (1.86) | 7.21 (1.80) | 7.31 (1.64) | 2.79 | .25 NS |
|  | **Trust** | 5.96 (1.97) | 6.18 (1.79) | 6.22 (2.12) | 2.47 | .29 NS |
| **Bill Shorten** | **Impression** | 7.09 (1.87) | 7.14 (1.79) | 7.11 (1.71) | 0.12 | .94 NS |
|  | **Likeability** | 6.70 (1.92) | 6.84 (1.84) | 7.01 (1.69) | 2.00 | .37 NS |
|  | **Trust** | 5.89 (2.10) | 5.99 (1.91) | 6.11 (2.18) | 1.90 | .39 NS |
