# Supplementary material for: The “multiple exposure effect” (MEE): How multiple exposures to similarly biased online content can cause increasingly larger shifts in opinions and voting preferences
Source: PLoS One. 2025 May 12;20(5):e0322900. doi: 10.1371/journal.pone.0322900 (PMC12068600; doi:10.1371/journal.pone.0322900)
Supplement: S19 Table — (DOCX) [file pone.0322900.s036.docx]

**S19 Table. Experiment 3: Pre- and post-exposure opinion ratings of Scott Morrison and Bill Shorten measured on a 10-point scale, control group only.**

|  | |  | **Scott Morrison Mean** (**SD)** |  | | **Bill Shorten Mean** (**SD)** |  | |  |  |
| --- | --- | --- | --- | --- | --- | --- | --- | --- | --- | --- |
|  |  | | **Pre** | **Post** | **Diff** | **Pre** | **Post** | **Diff** | ***z***^†^ | ***p*** |
| **1st Exposure** | **Impression** | | 7.13 (1.68) | 6.91 (1.92) | -0.22 | 7.11 (1.71) | 6.89 (1.94) | -0.22 | -0.51 | .61 NS |
|  | **Likeability** | | 7.31 (1.64) | 6.78 (1.96) | -0.53 | 7.01 (1.69) | 6.74 (1.91) | -0.27 | -1.21 | .23 NS |
|  | **Trust** | | 6.22 (2.12) | 6.20 (2.25) | -0.02 | 6.11 (2.18) | 6.27 (2.28) | 0.16 | -1.34 | .18 NS |
| **2nd Exposure** | **Impression** | | - | 6.57 (2.21) | -0.56 | - | 7.03 (1.83) | -0.08 | -1.94 | .05 NS |
|  | **Likeability** | | - | 6.51 (2.09) | -0.80 | - | 6.79 (1.86) | -0.22 | -3.01 | .003 |
|  | **Trust** | | - | 6.08 (2.33) | -0.14 | - | 6.30 (2.20) | 0.19 | -1.54 | .12 NS |
| **3rd Exposure** | **Impression** | | - | 6.48 (2.08) | -0.65 | - | 6.77 (1.95) | -0.34 | -1.26 | .21 NS |
|  | **Likeability** | | - | 6.43 (2.15) | -0.88 | - | 6.71 (1.92) | -0.30 | -2.55 | .01 |
|  | **Trust** | | - | 5.96 (2.27) | -0.26 | - | 6.23 (2.22) | 0.12 | -2.07 | .04 |

*Note*: The means from 2nd exposure and 3rd exposure are being compared to the pre-exposure mean.

^†^The z values come from Wilcoxon signed ranks test between post-exposure minus pre-exposure ratings for Scott Morrison and the post-exposure minus pre-exposure ratings for Bill Shorten.
